# Survival and quality of life in patients with lower risk myelodysplastic syndromes exposed to erythropoiesis-stimulating agents: an observational cohort study

**DOI:** 10.1016/S2352-3026(24)00350-8

**Published:** 2025-02-03

**Authors:** Hege Kristin Gravdahl Garelius, Timothy Bagguley, Adele Taylor, Pierre Fenaux, David Bowen, Argiris Symeonidis, Moshe Mittelmann, Reinhard Stauder, Jaroslav Čermák, Guillermo Sanz, Saskia Langemeijer, Luca Malcovati, Ulrich Germing, Laurence Sanhes, Maud d'Aveni, Dominic Culligan, Ioannis Kotsianidis, Karin A Koinig, Corine van Marrewijk, Simon Crouch, Theo deWitte, Alexandra Smith, Eva Hellström-Lindberg

**Affiliations:** a Sahlgrenska University Hospital, Gothenburg, Sweden; b Epidemiology & Cancer Statistics Group, Department of Health Sciences, University of York, York, UK; c Hôpital Saint-Louis, Paris, France; d Leeds Teaching Hospitals NHS Trust, Leeds, UK; e University of Patras School of Medicine, Patras, Greece; f Tel Aviv Sourasky Medical Center, Tel Aviv-Yafo, Israel; g Medizinische Universität Innsbruck, Innsbruck, Austria; h Department of Public Health, Health Services Research, and Health Technology Assessment, UMIT Tirol–The Tyrolean Private University, Hall in Tirol, Austria; i Institute of Hematology and Blood Transfusion, Prague, Czech Republic; j Consorcio Hospital General Universitario de Valencia, Valencia, Spain; k Radboud University Medical Center, Nijmegen, Netherlands; l University of Pavia & IRCCS Policlinico San Matteo, Pavia, Italy; m Universitätsklinikum Düsseldorf, Düsseldorf, Germany; n Centre Hospitalier de Perpignan, Perpignan, France; o Centre Hospitalier Régional Universitaire de Nancy Hôpitaux de Brabois, Vandèuvre-lès-Nancy, France; p Aberdeen Royal Infirmary, Aberdeen, UK; q General University Hospital of Alexandroupolis, Alexandroupoli, Greece; r Karolinska Institute, Solna, Sweden

## Abstract

**Background:**

In our previous study on erythropoiesis-stimulating agent (ESA) treatment in lower risk myelodysplastic syndromes from the European MDS (EUMDS) Registry, we showed that patients treated with ESAs had longer survival compared with patients who receive red blood cell transfusion (RBCT). In this study, with a longer follow up time and more patients included, we aimed to assess long-term effects on survival and health-related quality of life (HRQoL) of exposure to ESAs with or without RBCT in patients with lower risk myelodysplastic syndromes.

**Methods:**

The EUMDS Registry is a non-interventional, longitudinal, real-world registry prospectively enrolling newly diagnosed patients older than 18 years with lower risk (International Prognostic Scoring System low or intermediate-1) myelodysplastic syndromes from 16 European countries and Israel. The analysis was restricted to patients with haemoglobin concentrations less than 100 g/L enrolled between Jan 1, 2008, and July 1, 2019, with last censoring of data on Dec 31, 2021. Patient management was recorded every 6 months, including treatment, transfusions, and HRQoL. ESA treatment followed local guidelines. The patients were separated into four groups at each study visit: no ESA or RBCT, ESA only, ESA plus RBCT, and RBCT only. The data were analysed longitudinally over time according to ESA and RBCT status during each 6-month interval, using propensity score matching. The main outcomes were median overall survival and leukaemia-free survival, and HRQoL. This study is registered with ClinicalTrials.gov, NCT00600860, as is ongoing.

**Findings:**

2448 patients (the ESA-unexposed group [n=1265] and ESA-exposed group [n=1183]) were diagnosed before July 1, 2019; 1520 (62·1%) were male and 928 (37·9%) were female. Median follow-up time was 3·9 years (IQR 1·6–6·5). After applying eligibility criteria and propensity matching, there were 426 patients in the ESA-unexposed group and 744 patients in the ESA-exposed group. Median overall survival in the ESA exposed group was 44·9 months (95% CI 40·2–50·5) compared with 34·8 months (28·6–39·2) in the ESA unexposed group; the absolute difference was 10·1 months (95% CI 2·2–18·0; hazard ratio [HR] 0·70 [95% CI 0·59–0·83]; p<0·0001). Patients without RBCT in the presence or absence of ESA exposure maintained significantly better HRQoL than those with RBCT, irrespective of ESA exposure (linear mixed effect model of EQ-5d-3L index score, RBCT coefficient –0·04 [95% CI –0·06 to 0·03], p<0·0001; linear mixed effect model of VAS, –4·57 [–6·02 to –3·13], p<0·0001).

**Interpretation:**

ESA treatment in patients with lower risk myelodysplastic syndromes significantly improves overall survival when started before or early after the onset of regular transfusion therapy. Avoiding RBCT is associated with significantly better HRQoL.

**Funding:**

H2020 European Research Council, Novartis Pharmacy B V Oncology Europe, Amgen, BMS/Celgene International, Janssen Pharmaceutica, Takeda Pharmaceuticals International, and Gilead Sciences.

## Introduction

Erythropoiesis-stimulating agents (ESAs) are recommended as first-line treatment for anaemia in patients with myelodysplastic syndromes by European guidelines,^1^, ^2^ the National Comprehensive Cancer Network (NCCN) guidelines,^2^ and the Nordic myelodysplastic syndrome guidelines.^3^ ESA treatment was finally approved for use in lower risk myelodysplastic syndromes by EMA in 2017,^4^ after being used off-label for more than 20 years. The basis for the approval was two prospective randomised trials^5^, ^6^ comparing ESA versus placebo in lower risk myelodysplastic syndromes with haemoglobin less than 100 g/L and with no or low transfusion need. The studies showed that darbepoetin alfa and epoetin alfa significantly reduced transfusion need, but the studies were not designed or powered to assess survival or long-term outcome.^5^, ^6^ Two large epidemiological trials^7^, ^8^ indicated that exposure to ESAs is significantly associated with improved overall survival in patients with no or limited (<2 U/month) red blood cell transfusions (RBCTs) before ESA initiation, but the retrospective, real-world nature of these studies prevented solid conclusions. Several other phase 2 studies have shown reduced transfusion intensity and better health-related quality of life (HRQoL) with ESA treatment.^5^, ^6^, ^8^, ^9^, ^10^, ^11^ Based on these data on efficacy and HRQoL, a large systematic review by Park and Greenberg^12^ proposed ESAs as a first-line treatment for lower risk myelodysplastic syndromes.

**Research in context**

**Evidence before this study**

European and National Comprehensive Cancer Network (NCCN) guidelines recommend erythropoiesis-stimulating agents (ESAs) for patients with lower risk myelodysplastic syndromes and symptomatic anaemia. We searched PubMed from Jan 1, 2010, to Jan 15, 2023 for papers published in English using the search terms “lower risk myelodysplastic syndromes”, “treatment “, “erythropoiesis-stimulating factors” (ESAs), “red blood cell transfusion”, and “lower risk myelodysplastic syndrome” . Publications before 2010 were used when considered important from looking at reference lists of identified papers. The evidence that ESA treatment increases haemoglobin concentrations and prolong time to onset of a regular transfusion need is based on a published systematic literature review, numerous phase 2 studies and two prospective placebo-controlled phase 3 trials. No study has been designed to assess the effect ESA on overall survival and quality of life.

**Added value of this study**

The European MDS (EUMDS) Registry is a prospective, non-interventional, longitudinal, observational cohort study with a unique long-term analysis of a well controlled large cohort of newly diagnosed patients older than 18 years with lower risk (International Prognostic Scoring System low or intermediate-1) myelodysplastic syndromes from 16 European countries and Israel. The endpoints of overall survival and health-related quality of life were chosen to minimise investigators' subjective interpretation and encompass exposure to treatment, given transfusions, patient-reported quality of life, and death. It is a strength that ESA treatment was administered based on local routines only and that no subjective response assessment was performed. The study showed that patients exposed to ESA have significantly longer overall survival than those without ESA exposure, irrespective of treatment duration and response. ESA exposure was also associated with improved quality of life. The efficacy of ESA was better when initiated before or early after the onset of a permanent transfusion need.

**Implications of all the available evidence**

It is unlikely that a prospective placebo-controlled trial designed to assess long-term survival in patients with lower risk myelodysplastic syndromes will be performed. The present study provides evidence that ESA treatment prolongs survival and improves quality of life in patients with anaemia and myelodysplastic syndrome. The clinical implication of this study is that ESA treatment should be recommended at the onset of or early after start of symptomatic anaemia with or without transfusions. These data might influence policy and clinical practice in countries where ESAs are not available and in countries where patients are reimbursed only after the onset of a regular transfusion need.

The European Myelodysplastic Syndromes (EUMDS) Registry started in 2008 as a non-interventional longitudinal study, enrolling newly diagnosed patients with lower risk (International Prognostic Scoring System [IPSS] low or intermediate-1) myelodysplastic syndromes, currently involving 20 European countries and Israel. Participating centres include university clinics and larger regional hospitals.^13^ The patients are diagnosed and treated according to local, national, or international recommendations.

A report from the EUMDS Registry encompassing 1690 patients showed that 50% of patients had haemoglobin concentrations less than 100 g/L at baseline, and that more than 30% had received RBCT before diagnosis. HRQoL assessed by the EQ-5D-3L tool showed significantly lower overall scores and more frequent symptoms in patients with haemoglobin concentrations less than 100 g/L than in those with higher haemoglobin concentrations.^14^ In 2017, we reported a significantly longer time to start of regular transfusion therapy in patients exposed to ESA before the onset of a regular transfusion need.^9^

The present study was used to assess the effects of ESA exposure on overall survival and leukaemia-free survival and on quality of life over time.

## Methods

### Study design and participants

This study is registered with ClinicalTrials.gov, NCT00600860. The European MDS (EUMDS) Registry is a non-interventional, longitudinal, real-world registry prospectively enrolling patients from 149 centres in 16 countries across Europe and Israel. Patients eligible for inclusion were older than 18 years (with no upper age limit), with lower risk (IPSS low-risk or Intermediate-1 risk) myelodysplastic syndromes (within 3 months of diagnosis) as defined by the WHO classification 2016^15^ who were able and willing to provide written informed consent. Patients with higher risk myelodysplastic syndromes (as defined by the IPSS score) were excluded. Documented informed consent has been obtained for all patients before they were registered. The informed consent procedure was in accordance with the ICH guidelines on Good Clinical Practice (ICH-GCP) and with national and local regulatory requirements. The study protocol has been approved by the Local, Regional, or National Ethics Committees and has been published online. Patients for whom cytogenetic testing was not available or not successful could be included if the number of blasts was less than 5% and the cytopenia score was zero (ie, low risk).

### Procedures

Data were collected at baseline and at each 6-month outpatient routine follow-up visit in a central database. Information was collected on peripheral blood values, conventional iron parameters, bone marrow pathology, progression to higher risk myelodysplastic syndromes or acute myeloid leukaemia, Karnofsky status, comorbidities, transfusion history (including receipt and exact number of RBCTs), management of myelodysplastic syndromes, and start and stop dates for treatment (including use of ESAs). The revised IPSS (IPSS-R)^16^ was calculated from the component information recorded in the database. Patients were asked to complete an EQ-5D-3L questionnaire on HRQoL and mark their well-being according to a visual analog scale (VAS)^17^ at each visit as described in previous studies,^9^ as well as complete a written assessment from the physician on the patient's performance status. Patients were prospectively followed up until death, loss to follow-up, or withdrawal of informed consent. Importantly, actual interventions were decided by the physician in charge and follow national and local guidelines.

Biologicial sex was self-reported by patients and data on ethnicity and race were not collected. Patients were included from Jan 1, 2008, to July 1, 2019, and followed up until Dec 31, 2021 (last censoring). The analysis was restricted to patients with haemoglobin less than 100 g/L**.** Patients at the first visit who had their haemoglobin recorded as less than 100 g/L were considered to have reached the eligibility criteria. The patients in the initial study cohort who had a haemoglobin value of less than 100 g/L recorded were divided into two groups: ESA unexposed and ESA exposed. Patients in the ESA-exposed group were excluded from the analysis if they had started ESA after July 1, 2019 or before the definite diagnosis of myelodysplastic syndrome, also if they had started ESA treatment at their last recorded visit without follow-up data available or, if their haemoglobin value at start of ESA exposure had not been recorded. The same eligibility criteria were applied to the patients in the ESA-unexposed group.

Importantly, the registry does not record the treating physician's subjective view on whether a particular treatment resulted in a clinical response or not, meaning that the efficacy is assessed by the regularly reported variables only. In the present analysis, the main efficacy parameter was freedom or not from RBCT during the 6-month interval preceding each visit. The second efficacy parameter was HRQoL, also recorded at 6-month intervals. The patients are defined as exposed or not exposed to ESA, irrespective of efficacy of the given treatment.

To aid conclusions in this study, we performed a survey asking investigators about indications and reimbursement pattern for ESA treatment in their respective country during the period of the analysis (appendix p 23).

### Outcomes

The main outcomes were overall survival and progression to acute myeloid leukaemia, measured from the first visit a haemoglobin value of less than 100 g/L was recorded to date of death and acute myeloid leukaemia diagnosis, respectively. For patients in the ESA-exposed group, overall survival was measured from the start of ESA exposure, stratified by the number of RBCTs received before commencing ESA treatment (no RBCT, ≤4 red blood cell [RBC] units, or >4 RBC units).

HRQoL was measured using the EQ-5D-3L instrument for both the dimensions and VAS.

### Statistical analysis

Odds ratios (ORs) and their corresponding 95% CIs were estimated using logistic regression to examine the association with the patient's demographic and disease factors at time of diagnosis and whether they were subsequently exposed to ESA. To overcome potential confounding by non-random allocation of ESA exposure, propensity score matching was performed to ensure that patients who were ESA exposed and ESA unexposed had similar characteristics (appendix p 12). Briefly, the propensity of ESA exposure based on each patient's characteristics was calculated (ie, a propensity score) using logistic regression and the covariates were selected a priori. Weights were then calculated for everyone as the inverse probability of ESA exposure. A pseudo population in which covariates (confounders) were equally distributed across the ESA-unexposed and ESA-exposed groups was created by the application of these weights to the study population. The proportional hazard regression models comparing time-to-event outcomes in patients who were ESA exposed and ESA unexposed were weighted^18^ by stabilised inverse probability of treatment weights,^19^ based on the propensity of ESA exposure with no further adjustments included in the outcome models. Patients still alive at the time of the analysis were censored at their last follow-up visit, or, if applicable, the date a patient withdrew their informed consent. ESA exposure was analysed as a time-dependent covariate to reduce immortal time bias (patients in the ESA exposed group were analysed until their exposure to ESA).

EUMDS was designed to collect data prospectively and to ensure missing data was minimised. This included features on the website-based database to check the quality of the data and visits to the centres were made by the study monitor. However, for performance status (Karnofsky status), missingness was an issue and we imputed these values for 225 (17·6%) of 1278 patients using the mi command in Stata version 17 using the variables age at diagnosis, sex, country, HRQoL (VAS and EQ-5D-3L dimensions) and MDS-comorbidity index.

Only patients with comparable propensity scores were included to assess the primary outcome of overall survival to estimate the effects of ESA exposure on outcomes using standard time-to-event analyses; the analysis had 96% power, based on a log rank analysis, to detect a hazard ratio (HR) of 0·8 for overall and leukemia-free survival compared with patients who had not been exposed to ESA. 95% CIs were also used and p<0·05 indicated a statistically significant difference.

For the HRQoL analysis, the patients who were included in the propensity analyses and who had completed the EQ-5D-3L questionnaires at both the visit at which they had reached the eligibility criteria and the subsequent visits were included. The patients were separated into four groups at each clinical visit, depending on the treatment received in the interval leading up to that visit: no ESA or RBCT, ESA only, ESA plus RBCT, and RBCT only. The longitudinal EQ-5D-3L index and EQ-VAS were examined with linear mixed effect models,^20^ random intercept and slope, and with fixed effects used to determine whether HRQoL varied by treatment group (ESA and RBCTs) as well as to determine a linear trend over time. Additionally, a comparison was made between the patients with and without RBCT, irrespective of ESA exposure. All analyses were performed in Stata (version 17).

### Role of the funding source

The funders of the study had no role in study design, data collection, data analysis, data interpretation, or writing of the report.

## Results

**Figure 1:**
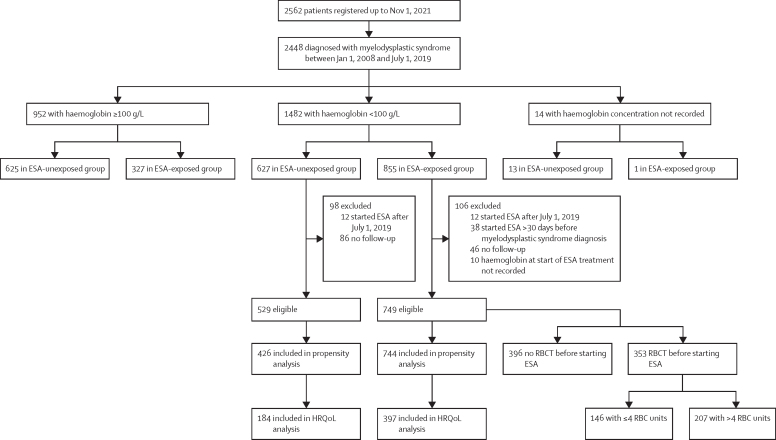
Study profile ESA=erythropoiesis-stimulating agent. HRQoL=health-related quality of life. RBC=red blood cell. RBCT=red blood cell transfusion.

Between Jan 1, 2008, and July 1, 2019, 2448 patients (study cohort**;** 1520 [62·1%] were male and 928 [37·9%] were female) were diagnosed and included in the registry; with a median follow-up of 3·9 years (IQR 1·6–6·5). In 952 (38·9%) of 2448 patients, haemoglobin concentrations were greater than or equal to 100 g/L (figure 1), and the overall survival for these patients compared with those who had haemoglobin concentrations less than 100 g/L at one or more timepoints (1482 [59·5%]), is shown in the appendix (p 2), with haemoglobin treated as a time-varying covariate.^21^ The 14 patients with their haemoglobin concentration not recorded were excluded from this analysis due to missing data.

Patient characteristics in the initial study cohort, separated into all ESA-exposed (n=1183) and all ESA-unexposed (n=1265) groups, were reported (table). The distribution of haemoglobin concentrations before ESA exposure in the participating countries are shown in the appendix (p 3), with an overall median haemoglobin concentration of 94 g/L (IQR 42–151). 108 (9·1%) patients in the ESA-exposed group and 51 (4·1%) patients in the ESA-unexposed group received lenalidomide, and four (0·3%) in the ESA-exposed group and one (0·1%) patient in the ESA-unexposed group received luspatercept.

**Table 1:** Baseline characteristics of patients by exposure to ESA and the probability of exposure to ESA in an univariable logistic regression model

		**Total N (%)**	**ESA-exposured group**	**ESA-unexposed group**	**Odds ratio (95% CI)**	**p value**
Total		2448 (100%)	1183 (100%)	1265 (100%)	..	..
Age at diagnosis, years		..	..	..	..	<0·0001
	<60	247 (10·1%)	94 (7·9%)	153 (12·1%)	0·58 (0·44– 0·76)	..
	60–74	992 (40·5%)	465 (39·3%)	527 (41·7%)	0·83 (0·70– 0·98)	..
	75+	1209 (49·4%)	624 (52·7%)	585 (46·2%)	1 (reference)	..
Sex		..	..	..	..	0·0050
	Male	1520 (62·1%)	701 (59·3%)	819 (64·7%)	1 (reference)	..
	Female	928 (37·9%)	482 (40·7%)	446 (35·3%)	1·26 (1·07–1·49)	..
MDS diagnosis		..	..	..	..	<0·0001
	Refractory anaemia	416 (17·0%)	233 (19·7%)	183 (14·5%)	1·78 (1·41– 2·25)	..
	Refractory anaemia with ring sideroblasts	364 (14·9%)	227 (19·2%)	137 (10·8%)	2·32 (1·81– 2·97)	..
	Refractory cytopenia with multilineage dysplasia	984 (40·2%)	410 (34·7%)	574 (45·4%)	1 (reference)	..
	Refractory cytopenia with multilineage dysplasia and ring sideroblasts	146 (6·0%)	89 (7·5%)	57 (4·5%)	2·19 (1·53–3·12)	..
	Refractory anaemia with excess blasts-1 or refractory anaemia with excess blasts -2	299 (12·2%)	117 (9·9%)	182 (14·4%)	0·90 (0·69–1·17)	..
	Myelodysplastic syndrome, unclassifiable	97 (4·0%)	30 (2·5%)	67 (5·3%)	0·63 (0·40–0·98)	..
	5q-syndrome	142 (5·8%)	77 (6·5%)	65 (5·1%)	1·66 (1·16– 2·36)	..
Revised International Prognostic Scoring System risk category						
	Very low	613 (25·0%)	237 (20·0%)	376 (29·7%)	0·49 (0·40– 0·60)	<0·0001
	Low	1062 (43·4%)	596 (50·4%)	466 (36·8%)	1 (reference)	..
	Intermediate	412 (16·8%)	205 (17·3%)	207 (16·4%)	0·77 (0·62–0·97)	..
	High or very high	88 (3·6%)	37 (3·1%)	51 (4·0%)	0·57 (0·37–0·88)	..
	Unknown	273 (11·2%)	108 (9·1%)	165 (13·0%)	..	..
Country		..	..	..	..	<0·0001
	Austria	153 (6·3%)	56 (4·7%)	97 (7·7%)	0·47 (0·33–0·69)	..
	Croatia	18 (0·7%)	1 (0·1%)	17 (1·3%)	0·05 (0·01–0·37)	..
	Czech Republic	139 (5·7%)	50 (4·2%)	89 (7·0%)	0·46 (0·31–0·68)	..
	Denmark	61 (2·5%)	42 (3·6%)	19 (1·5%)	1·82 (1·03–3·21)	..
	France	510 (20·8%)	280 (23·7%)	230 (18·2%)	1 (reference)	..
	Germany	63 (2·6%)	19 (1·6%)	44 (3·5%)	0·35 (0·20–0·62)	..
	Greece	333 (13·6%)	185 (15·6%)	148 (11·7%)	1·03 (0·78–1·36)	..
	Israel	208 (8·5%)	108 (9·1%)	100 (7·9%)	0·89 (0·64–1·23)	..
	Italy	93 (3·8%)	44 (3·7%)	49 (3·9%)	0·74 (0·47–1·15)	..
	Netherlands	96 (3·9%)	47 (4·0%)	49 (3·9%)	0·79 (0·51–1·22)	..
	Poland	55 (2·2%)	19 (1·6%)	36 (2·8%)	0·43 (0·24–0·78)	..
	Portugal	38 (1·6%)	20 (1·7%)	18 (1·4%)	0·91 (0·47–1·77)	..
	Romania	44 (1·8%)	27 (2·3%)	17 (1·3%)	1·30 (0·69–2·45)	..
	Serbia	28 (1·1%)	1 (0·1%)	27 (2·1%)	0·03 (0·00–0·23)	..
	Spain	135 (5·5%)	73 (6·2%)	62 (4·9%)	0·97 (0·66–1·42)	..
	Sweden	107 (4·4%)	72 (6·1%)	35 (2·8%)	1·69 (1·09–2·62)	..
	UK	367 (15·0%)	139 (11·7%)	228 (18·0%)	0·50 (0·38–0·66)	..

Data are n (%) unless otherwise specified. Race or ethnicity is not a variable in the European MDS Registry because in some European countries it is not allowed to be recorded, and the definition differs by each country. ESA=erythropoiesis-stimulating agent.

After applying eligibility criteria, 529 patients in the ESA unexposed group and 749 patients in the ESA-exposed group were eligible for analysis, of whom 426 (80·5%) in the ESA-unexposed group and 744 (99·3%) in the ESA-exposed group had comparable propensity scores (figure 1; appendix p 9). These patients were selected to estimate the effects of ESA on overall survival and leukaemia-free survival. The same groups were also used in the HRQoL analysis, of whom 184 (43·2%) of 426 ESA patients unexposed to ESA and 397 (53·4%) of 744 patients exposed to ESA had completed a questionnaire at both visits 1 and 2 after fulfilling the eligibility criteria. There were no differences in the baseline characteristics at the time of meeting the eligibility criteria between those who did complete the two questionnaires versus those who did not (appendix p 15). Median length of time on ESA was 15·4 months (IQR 6·6–29·9) for patients without RBCT before treatment and 11·4 months (5·3–24·2) for patients with RBCT before starting ESA. The median length of time on ESA for patients receiving ≤4 RBC units and >4 RBC units was 14·7 months (IQR 6·5–29·4) and 10·2 months (4·7–20·1), respectively.

**Figure 2:**
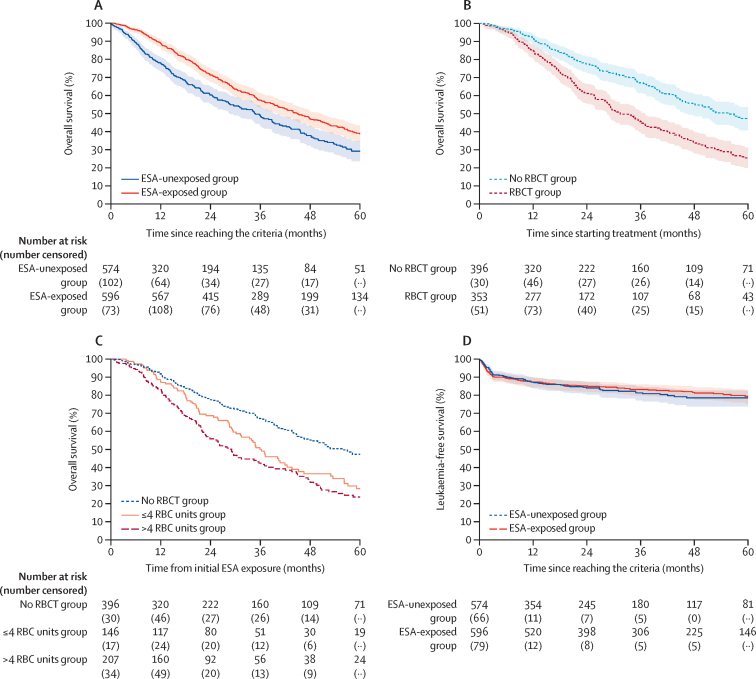
Overall survival and leukaemia-free survival by treatment status (A) Inverse probability of treatment weighted Kaplan–Meier curve for overall survival: median overall survival of patients in the ESA-exposed (time-varying covariate) group (44·9 months [40·2–50·5]) *vs* ESA-unexposed group (34·8 months [28·6–39·2]). (B) Overall survival of patients in the ESA-exposed group stratified by RBCT status before ESA exposure (p<0·0001). (C) Overall survival of the ESA-exposed group based on the number of pre-exposure transfusions given; no RBC units (n=396), 0–4 RBC units (n=146), and >4 RBC units (n=207). (D) Inverse probability of treatment weighted Kaplan–Meier curve for time to acute myeloid leukaemia transformation by ESA exposure (p=0·86). ESA=erythropoiesis-stimulating agent. RBC=red blood cell. RBCT=red blood cell transfusion.

668 (57·1%) of 1170 patients included in the survival analysis had died. Median overall survival from the time of reaching the eligibility criteria in the ESA exposed group versus ESA-unexposed group was 44·9 months (95% CI 40·2–50·5) and 34·8 months (28·6–39·2), respectively (HR 0·70 [95% CI 0·59–0·83]; p<0·0001; figure 2A). The analysis was repeated excluding patients with refractory anaemia with ring sideroblasts (n=205) and refractory cytopenia with multilineage dysplasia and ring sideroblasts (n=78) and the findings were similar (HR 0·69 [95%CI 0·57–0·83]; p<0·0001). In the ESA-exposed group, 402 (53·7%) of 749 patients had died. Patients without RBCT need before ESA exposure (figure 2B) had a median overall survival of 55·9 months (95% CI 46·6–64·3) and in those with RBCT before ESA the median overall survival was 31·4 months (28·6–37·9; p<0·0001). Overall survival was worse for those patients who had RBCT before commencing ESA (HR 1·85 [95% CI 1·50–2·89]; p<0·0001); this association remained significant after adjustment for the characteristics (age, sex, revised International Prognostic Scoring System, Karnofsky status, co-morbidity index, bone marrow blast, and haemoglobin concentration; HR 1·51 [95% CI 1·20–1·90]; appendix p 18). Survival analysis of the ESA-exposed group was further divided based on the cumulative number of pre-exposure transfusions received: no RBCT (n=396), less than 4 RBC units (n=146), and 4 or more units (n=207; figure 2C). 5-year overall survival was 47·3% (95% CI 40·7–53·6) in the group with no RBCT, 28·3% (19·4–37·8) in the group with less than 4 RBC units, and 23·7% (16·8–31·2) in the group with 4 or more RBC units. Patients receiving 4 or more RBC units before commencing ESA had a poorer overall survival compared with those who had received less than 4 RBC units (HR 1·35 [95% CI 1·01–1·80]; adjusted HR 1·22 [0·91–1·66]; p=0·188). There was no significant difference in acute myeloid leukaemia transformation between the ESA-exposed group (130 [17·5%] of 744 patients) and the ESA-unexposed group (78 [18·3%] of 426 patients; figure 2D).

Another important aspect of early ESA treatment is to prevent or delay the onset of a regular RBCT need, which was shown in an earlier study.^9^ Patients who already had RBCT before ESA exposure were more likely to receive a post-ESA RBCT (HR 2·30 [95% CI 1·93–2·73]; p<0·0001); less than 4 RBC units: 1·45 [1·15–1·81]; 4 or more RBC units: 3·63 [2·97–4·43]). Overall, the median time to first RBCT after ESA exposure was 7·5 months (95% CI 6·0–9·5)**,** and median time to first transfusion after ESA exposure was 16·0 months (95% CI 12·3–21·2) in patients without RBCT, 9·1 months (5·8–12·5) in those with less than 4 RBC units, and 3·4 months (2·7–4·2) in those with 4 or more RBC units before ESA exposure. The percentage of patients never reaching transfusion independence at 6 months was 41·8% (61 of 146 patients) in the group with less than 4 RBC units and 78·4% (163 of 207 patients) in the group with 4 or more RBC units.

The EUMDS Registry does not mandate serum erythropoietin concentrations to be assessed, only to be reported when clinically assessed. Since this assessment was not available for most patients (315 [60%] of 1278 patients), we did not include this parameter in our analysis. Moreover, although start and stop dates for ESA treatment were reported, the information on the exact dose of ESA was not captured, as mandatory from the Registry; therefore, this measure was available only for a minority (71 [10%] of 749 patients) of patients.

**Figure 3:**
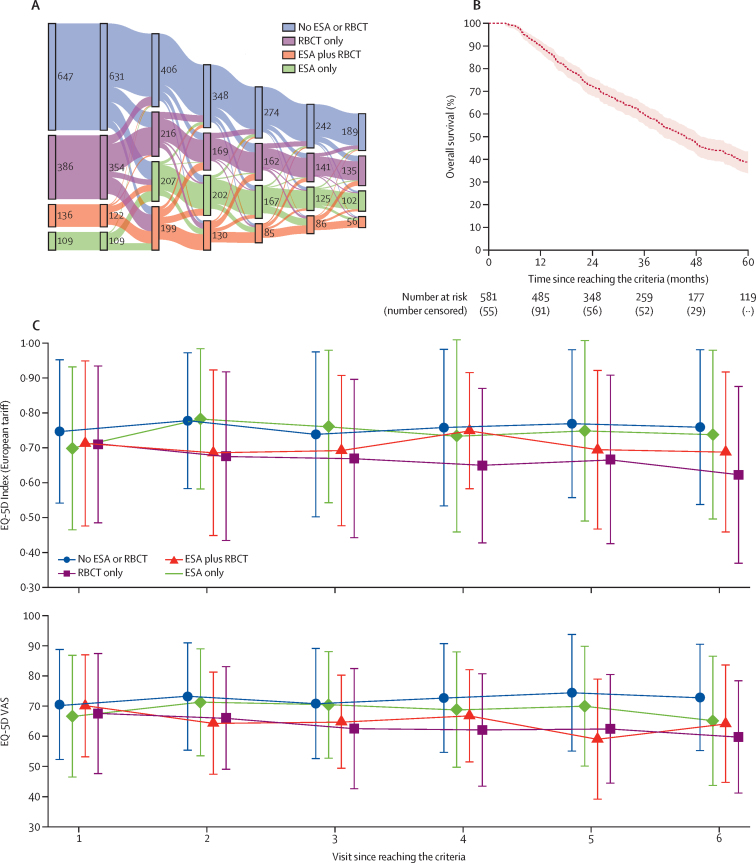
Health-related quality of life by treatment status (A) Sankey diagram illustrating the flow of patients between the four groups. (B) Overall survival curves for patients included in the HRQoL analysis.(C) Mean HRQoL by ESA or RBCT status over time since reaching the eligibility criteria. RBCT and ESA status was defined at each visit. ESA=erythropoiesis-stimulating agent. HRQoL=health-related quality of life. RBCT=red blood cell transfusion. VAS=Visual Analog Scale.

The Sankey diagram shows the number of patients at each visit evaluated for treatment with RBCT or ESA; 647 (50·6%) of 1278 patients had received neither at visit 1 (figure 3A). Median survival was 45·4 months (95% CI 40·4–49·1; figure 3B). HRQoL was examined up to visit 6 for the 581 patients who had completed a questionnaire at both visit 1 and 2 (figure 3C). Patients who did not require ESA or RBCT reported, on average, the highest mean HRQoL. By contrast, patients who received RBCT had the lowest HRQoL (linear mixed effect model of EQ-5d-3L index score, RBCT coefficient –0·04 [95% CI –0·06 to 0·03], p<0·0001; linear mixed effect model of VAS, –4·57 [–6·02 to –3·13], p<0·0001). Importantly, for patients exposed to ESA who remained RBCT-free, HRQoL did not deteriorate over time (Index upper curve p=0·014; VAS, lower curve=0·207; figure 3C). Patients, on average, completed four questionnaires. There was no marked difference in the completion rate nor in the baseline characteristics (appendix p 20) by baseline treatment group, meaning that all groups were comparable.

## Discussion

The EUMDS Registry, which has enrolled patients since 2008, provides a unique possibility to assess the long-term effects of interventions given as part of real-life management of patients with myelodysplastic syndromes. As the registry covers a broad range of European countries, it also provides a possibility to compare different health-care strategies and, indirectly, reimbursement systems. To avoid subjective interpretations by the treating physicians, “response to treatment” is not a recorded variable, whereas exposure to treatment, and freedom from RBCT and HRQoL, are systematically recorded in detail during a patient's clinical course. To compare intervention strategies, outcomes in different cohorts were compared using propensity-score matching.^18^, ^19^

ESAs are the most frequently applied treatments for anaemia in lower risk myelodysplastic syndromes. Interestingly, anaemia proved to be the cytopenia with the most adverse prognostic impact in the new International Prognostic Scoring System-Molecular prognostic system.^22^ RBCT need was not recorded in the IPSS-M cohort; however, a recently published Swedish IPSS-M sub-study showed that transfusion state at 8 months, reflecting presence or absence of RBCTs between 4 months and 8 months after diagnosis, is an independent predictor of overall survival with the IPSS-M score.^23^ The heterogeneous international reimbursement pattern for ESAs leads to different treatment patterns for symptomatic or transfusion-dependent anaemia across countries.

It is well-established that anaemia and in particular transfusion need in myelodysplastic syndromes is associated with impaired survival and quality of life, as well as that ESA treatment prevents or reduces the frequency of transfusions and improves quality of life.^5^, ^9^, ^24^ However, these facts do not allow for the interpretation that intervention with ESA prolongs overall survival, partly as reasons for anaemia-associated reduced survival in myelodysplastic syndromes are multifactorial and incompletely understood. In a previous study analysing the EUMDS Registry, we showed that exposure to ESA treatment before the onset of transfusion dependence significantly prolonged time to first transfusion event compared with treatment starting after the onset of a transfusion need.^9^

The present study was designed and powered to assess the effects of ESA exposure on long-term outcome. Our findings show a significant survival advantage for patients with lower risk myelodysplastic syndromes exposed to ESAs at the onset of substantial anaemia before transfusion therapy, compared with patients in the same clinical situation who were unexposed to ESA. The effect of treatment in patients with a low transfusion need (<4 RBC units) was better than in those with higher transfusion requirement but worse than for patients without transfusion. Additionally, ESA exposure was associated with higher HRQoL and over time with maintained HRQoL. By contrast, development of RBCT dependence was associated with significantly lower HRQoL, irrespective of ESA treatment.

Our data supports the conclusion that patients with lower risk myelodysplastic syndromes with substantial anaemia (haemoglobin concentration <100 g/L) fare significantly better both in terms of overall survival and HRQoL when exposed to ESA treatment, hence the chance to avoid or delay transfusion therapy. Our results indicate that ESA exposure should preferably start before a transfusion need or at least after a few transfusions. Naturally, the exact haemoglobin concentration provoking symptoms differ between patients, and this is why the actual cutoff was set to a level when a majority of patients have symptoms of anaemia.^14^

To date, the EUMDS Registry does not prove the efficacy of second-line alternatives over long-term follow-up (eg, lenalidomide, luspatercept, Roxadustat, and possibly allogeneic stem cell transplantation) but might serve as a useful source of data for second-line treatment in the future.

Limitations of this study encompass its design, specifically that this study was not a prospective randomised placebo-controlled trial and used heterogeneous patient material. At the same time, the heterogeneity of the patients underscores the fact that this material is more like real life than a randomised study. We have already argued that a sufficiently large randomised controlled trial powered to assess long-term survival will likely not be performed for a treatment that has been in clinical use for more than 30 years. The propensity scoring technique is a well established alternative to account for differences in characteristics between the treatment groups. We cannot exclude the possibility of some residual confounding. However, considering the size of the effect it is highly unlikely that residual confounding would explain the differences between patients treated with ESA and those not treated with ESA, and the large catchment area of the patient population could be viewed as an asset. The absence of certain variables, such as serum erythropoietin could also be criticised. However, we are not aiming to refine existing predictive scores for ESA treatment, rather we are recommending that treatment is initiated before the onset of a regular transfusion need.

Does this large European analysis provide a sufficient basis for the national authorities to approve or reimburse ESA treatment for patients with anaemia with myelodysplastic syndromes before they become transfusion dependent? The treatment is applied depending on local guidelines and reimbursement policies, and differences in patient profiles are compensated for by well documented propensity scoring methodology. We conclude that ESA exposure appears to provide a benefit for patients with lower risk myelodysplastic syndromes - by significantly delaying the onset of permanent transfusion dependency. Moreover, ESA exposure was also associated with improved overall survival and quality of life. Additionally, to our knowledge, neither this large registry analysis nor any previous phase 2 or phase 3 trial have given any safety concern of ESA treatment. While direct adverse events from treatment are not reported in the registry, long-term safety signals, including acute myeloid leukaemia transformation and death, are recorded and were improved overall. We propose that these data provide a basis for world-wide authorisation of ESA treatment for patients with lower risk myelodysplastic syndromes and symptomatic anaemia, irrespective of transfusion need.

### Contributors

### Data sharing

The Article is based on data from the European MDS Registry. The data are not publicly available due to privacy or ethical restrictions. Access to data that support the findings of this study can be obtained from the EUMDS project management office upon reasonable request from the EUMDS Research group in Nijmegen (mds-right@radboudumc.nl). A fee might be required.

## Declaration of interests

RS reports honoraria from BMS for the MDS Right Horizon 2020 Project; financial support for meetings from Celgene/BMS, Lilly, Teva, AbbVie, and AHOP; advisory board participation for BMS and Otsuka; and is Servier President of Verein Senioren Krebshilfe. CvM and TdW work for the EUMDS Registry, which is supported by an educational grant from Novartis Pharmacy BV Oncology Europe, Amgen, BMS, Janssen Pharmaceutical, Takeda Pharmaceutical, Gilead Sciences, and a European commission H2020 Grant. AS is supported in part by the National Institute for Health and Care Research (NIHR) Leeds Biomedical Research Centre (NIHR203331). HKGG, TB, AT, PF, DB, ArS, JC, SL, LM, UG, LS, MdA, DC, KAK, SCr, and EHL declare no competing interests.
