# Correction to *Lancet Haematol* 2025; 12: e128–37

**DOI:** 10.1016/S2352-3026(25)00076-6

**Published:** 2025-03-31

*Garelius HKG, Bagguley T, Taylor A, et al. Survival and quality of life in patients with lower risk myelodysplastic syndromes exposed to erythropoiesis-stimulating agents: an observational cohort study.* Lancet Haematol *2025; **12:** e128–37*—In this Article, the affiliation of the authors Reinhard Stauder and Karin A Koinig should have been “Department of Internal Medicine V (Hematology and Oncology), Innsbruck Medical University, Innsbruck, Austria”. Additionally, the other affiliation for Reinhard Stauder has been removed. These corrections have been made March 31, 2025.

