# Supplementary material for: Survival and quality of life in patients with lower risk myelodysplastic syndromes exposed to erythropoiesis-stimulating agents: an observational cohort study
Source: Lancet Haematol. 2025 Feb 3;12(2):e128–37. doi: 10.1016/S2352-3026(24)00350-8 (PMC11803517; doi:10.1016/S2352-3026(24)00350-8)
Supplement: Supplementary appendix [file mmc1.pdf]

THE LANCET
Haematology

Supplementary appendix

This appendix formed part of the original submission and has been peer reviewed. We post it as supplied by the authors.

Supplement to: Gravdahl Garelius HK, Bagguley T, Taylor A, et al. Survival and quality of life in patients with lower risk myelodysplastic syndromes exposed to erythropoiesis-stimulating agents: an observational cohort study. *Lancet Haematol* 2025; **12:** e128–37.

Supplementary Material

Contents

Supplementary Methods......1

Supplementary Tables and Figures......2

Figure S1: Overall survival by time-varying (4) levels of haemoglobin (<100 g/L vs. ≥100 g/L)......2

Figure S2: Box & Whisker Plot showing haemoglobin values prior to starting of exposure to erythropoietin-stimulating agents (ESA) by country......3

Table S1: Baseline characteristics of eligible patients (haemoglobin <100 g/L) by exposure to erythropoietin-stimulating agents (ESA) and the association between each factor and the probability of ESA exposure in an univariable logistic regression model......4

Table S2: Baseline characteristics of eligible patients (haemoglobin <100 g/L) by exposure to erythropoietin-stimulating agents (ESA) and the association between each factor and the probability of ESA exposure in a multivariable logistic regression model......7

Figure S3: Distribution of propensity score across exposed and unexposed groups......9

Table S3: Baseline characteristics of patients included in the propensity analysis by exposure to erythropoietin-stimulating agents (ESA) and the association between each factor and the probability of ESA exposure in a multivariable logistic regression model......10

Table S5: EQ-5D questionnaires completed at visits 1 & 2 after meeting the criteria by baseline characteristics and included in the propensity analysis stratified by ESA exposure......15

Table S6: Characteristics by Transfusion Status Prior to ESA exposure......18

Table S7: Exposed group at visit 1 after reaching the criteria for patients that completed EQ-5D questionnaires at visits 1 & 2 by baseline characteristics and included in the propensity analysis......20

Table S8: Reimbursement practice - personal information......23

Table S9: List of EUMDS participants......24

References 27

Supplementary Methods

The association with the patient's demographic and disease factors at time of diagnosis and whether they were subsequently exposed to ESA was estimated using univariable logistic regression. The derived categories were based on standard clinical usage. The impact of ESA exposure on overall survival was examined in patients who had haemoglobin (Hb) value of <100 g/L recorded at a visit, accordingly, patients were excluded if their recorded Hb values were always ≥100 g/Lor unknown ESA-exposed patients were also excluded if they had started ESA after 1st July 2019 or prior to being diagnosed with MDS, or they had only started treatment at their last recorded visit and so no follow-up data were available. Similar criteria around follow-up were applied to patients who were not exposed to ESA.

Overall survival was estimated using standard time-to-event analyses namely Kaplan-Meier and Cox's proportional hazard. However, in order to make a comparison in survival between those patients who were exposed to ESA versus those that were not, a statistical technique called inverse probability of treatment weighting (IPTW) was implemented, as we have previously reported (1). This technique considers the differences in the characteristics between treated and non-treated patients thus controlling for potential confounders allowing for a fair comparison between the groups. Firstly, the propensity of ESA exposure based on each patient's characteristics was calculated (i.e., a propensity score) using univariable logistic regression, these covariates were selected a priori, weights were then calculated for each individual as the inverse probability of receiving ESA. A pseudo-population in which covariates (confounders) are equally distributed across the ESA and non-ESA exposed groups was created by the application of these weights to the study population. The proportional hazards regression models comparing time-to-event outcomes in exposed and unexposed patients were weighted (2) by stabilized inverse probability of treatment weights (3) based on the propensity of ESA exposure with no further adjustments included in the outcome models. The Stata command pbalchk was used to check the balance. ESA exposure was analysed as a time-dependent covariate to reduce immortal time bias (ESA exposed patients were analysed in the unexposed group until they were exposed to ESA).

Table S1 describes the baseline characteristics amongst the eligible patients (Figure 1; n=529+749=1278), and the propensity to being exposed to ESA among eligible patients was modelled using logistic regression including demographic and disease-related factors identified a priori that influenced the decision to expose a patient to ESAs. All variables listed in Table S1 were included in the propensity model except for serum erythropoietin which was excluded due to high proportion of patients where this parameter was not measured (59.5%) and this differed by whether a patient received ESA. Covariates were fitted as a categorical variable in the model with the exception of age at diagnosis, the time between diagnosis to reaching the criteria and the haemoglobin at the time of reaching the criteria for exposure, these were all fitted as b-splines. Table S2 reports the outcome of the multivariable logistic regression.

Restricting the data to exposed and unexposed patients with comparable propensity scores resulted in the loss of only 108 patients, with 1,170/1,278 eligible patients (744 ESA exposed and 426 unexposed) included in the propensity model group (Figure S3, Table S3). Weighted comparisons of covariates in the propensity model (Table S4, bold column) showed no differences between ESA-exposed and unexposed patients and it was this dataset used to assess overall and leukaemia-free survival.

All analyses were performed in Stata version 17.

Table S8 shows the different reimbursement policies in the countries participating in the study.

Supplementary Tables and Figures

Figure S1: Overall survival by time-varying (4) levels of haemoglobin (<100 g/L vs. ≥100 g/L)

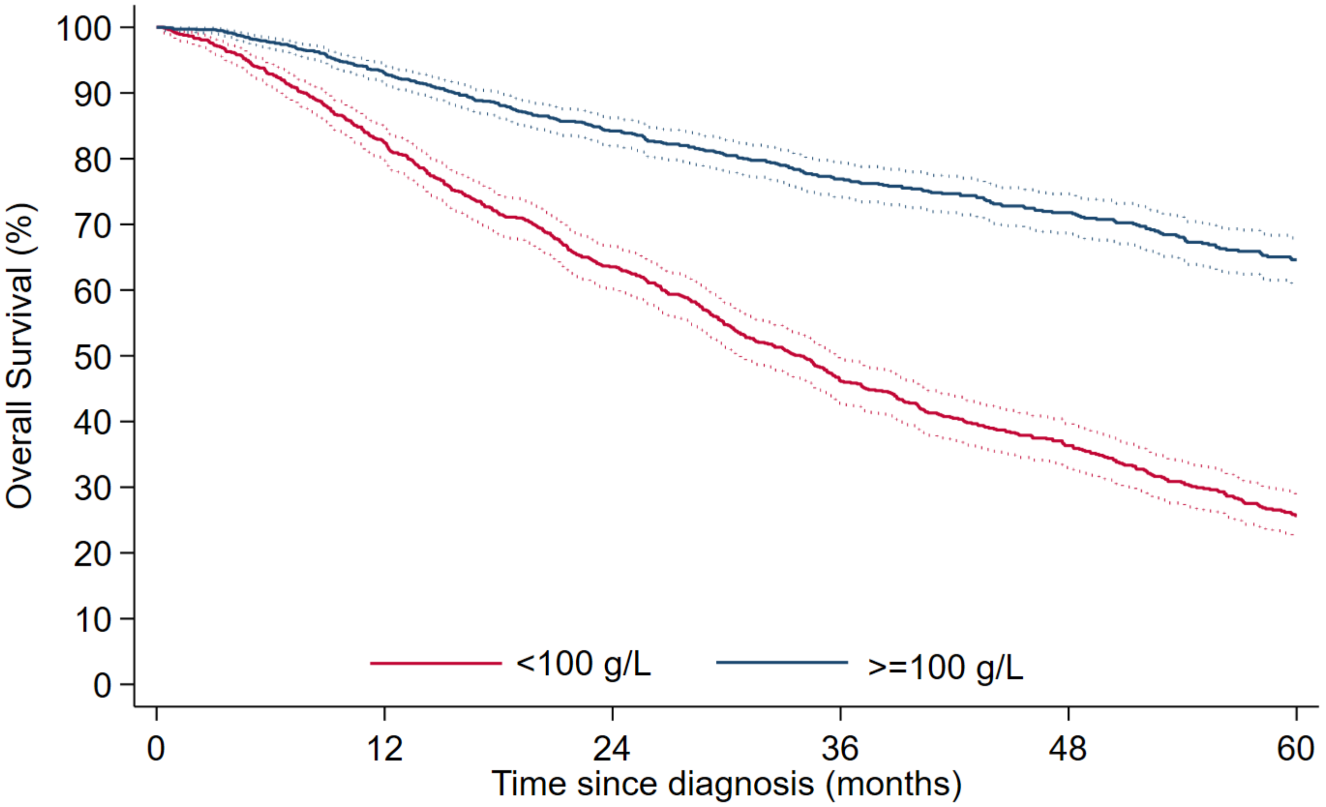

Figure S2: Box & Whisker Plot showing haemoglobin values prior to starting of exposure to erythropoietin-stimulating agents (ESA) by country

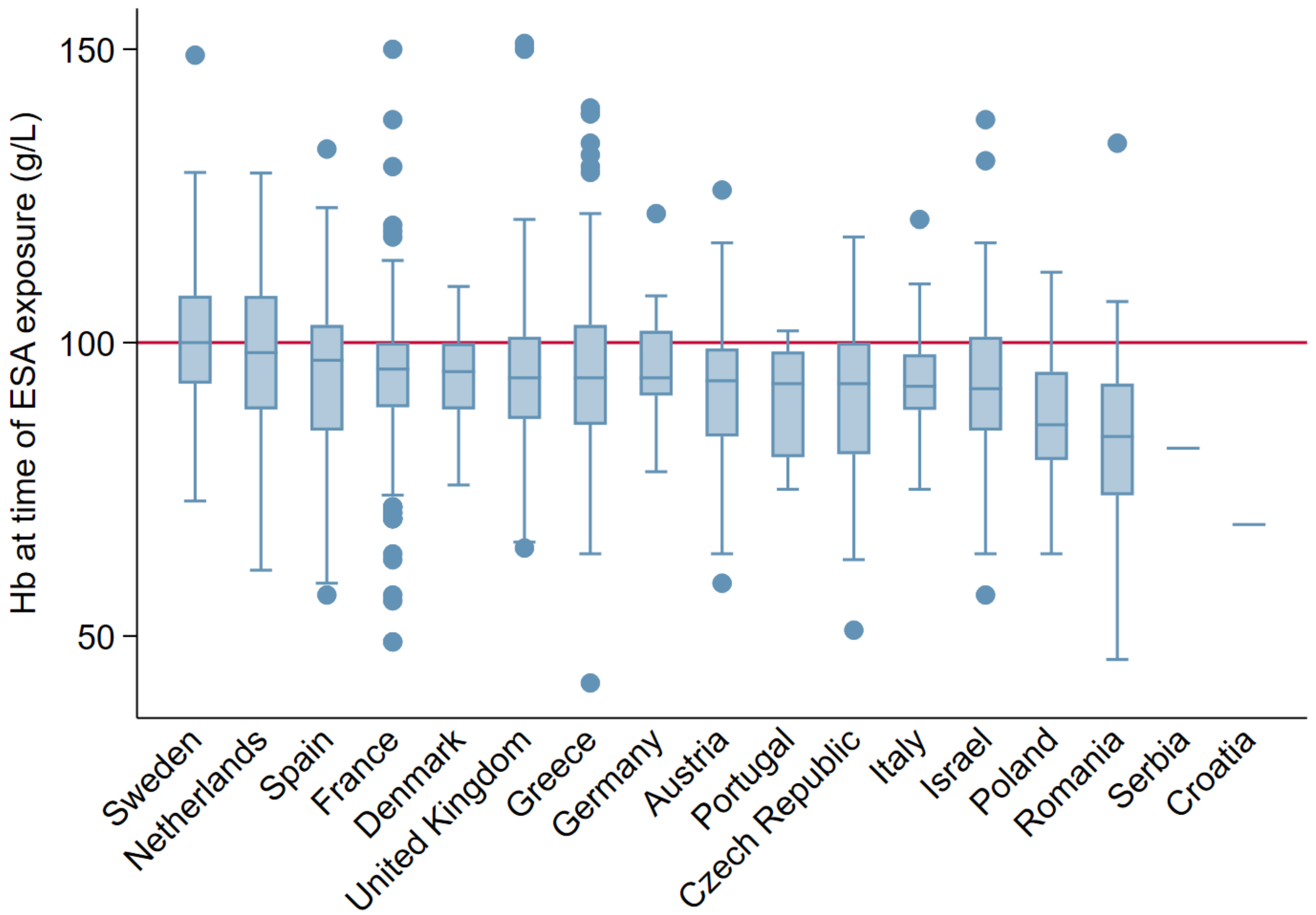

Table S1: Baseline characteristics of eligible patients (haemoglobin <100 g/L) by exposure to erythropoietin-stimulating agents (ESA) and the association between each factor and the probability of ESA exposure in an univariable logistic regression model

	Total N (%)	ESA Exposed N (%)		Odds Ratio (95% CI)	p-value
		Yes	No		
**Total**	1278 (100)	749 (58.6)	529 (41.4)		
**Age at Diagnosis (years)**					
<60	117 (9.2)	55 (7.3)	62 (11.7)	0.58 (0.39-0.86)	0.02
60-74	533 (41.7)	314 (41.9)	219 (41.4)	0.94 (0.74-1.18)	
75+	628 (49.1)	380 (50.7)	248 (46.9)	1 (ref)	
**Sex**					
Male	774 (60.6)	449 (74.7)	325 (48)	1 (ref)	0.59
Female	504 (39.4)	300 (49.9)	204 (30.1)	1.06 (0.85 - 1.34)	
**MDS diagnosis**[1]					
RA	203 (15.9)	141 (23.5)	62 (9.2)	2.09 (1.48 - 2.96)	<0.001
RARS	215 (16.8)	154 (25.6)	61 (9)	2.32 (1.64 - 3.28)	
RCMD	499 (39)	260 (43.3)	239 (35.3)	1 (ref)	
RCMD-RS	84 (6.6)	56 (9.3)	28 (4.1)	1.84 (1.13 - 2.99)	
RAEB-1 or RAEB-2	153 (12)	70 (11.6)	83 (12.3)	0.78 (0.54 - 1.11)	
MDS-U	37 (2.9)	17 (2.8)	20 (3)	0.78 (0.4 - 1.53)	
5q-syndrome	87 (6.8)	51 (8.5)	36 (5.3)	1.3 (0.82 - 2.07)	
**IPSS-R risk category**[2]					
Very low	46 (3.6)	29 (4.8)	17 (2.5)	0.9 (0.48 - 1.67)	<0.001
Low	665 (52)	436 (72.5)	229 (33.8)	1 (ref)	
Intermediate	308 (24.1)	172 (28.6)	136 (20.1)	0.66 (0.5 - 0.88)	
High/very high	107 (8.4)	41 (6.8)	66 (9.7)	0.33 (0.21 - 0.5)	
Unknown	152 (11.9)	71 (11.8)	81 (12)	0.46 (0.32 - 0.66)	
**Bone marrow blasts**[1]					0.01
<5%	1114 (87.2)	668 (111.1)	446 (65.9)	1 (ref)	
5-10%	164 (12.8)	81 (13.5)	83 (12.3)	0.65 (0.47 - 0.91)	
**Haemoglobin (g/L)**					0.20
80-100	1057 (82.7)	628 (83.8)	429 (81.1)	1 (ref)	
<80	221 (17.3)	121 (16.2)	100 (18.9)	0.83 (0.62-1.11)	
**Serum erythropoietin iU/L**					<0.0001
<100	95 (18.0)	222 (29.6)	95 (18.0)	1 (ref)	
100-500	75 (14.2)	136 (18.2)	75 (14.2)	0.78 (0.54-1.12)	
>500	44 (8.3)	31 (4.1)	44 (8.3)	0.30 (0.18-0.51)	
Not measured	315 (59.5)	360 (48.1)	315 (59.5)	-	

	Total N (%)	ESA Exposed N (%)		Odds Ratio (95% CI)	p-value
		Yes	No		
**Transfusions prior to reaching the criteria**					
No	675 (52.8)	360 (48.1)	284 (41.9)	1 (ref)	0.04
Yes	550 (43)	305 (50.7)	245 (36.2)	0.8 (0.64 - 1)	
**Time from diagnosis to reaching the criteria (months)**					
<6	979 (76.6)	629 (84.0)	350 (66.2)	1 (ref)	<0.0001
6-12	112 (8.8)	49 (6.5)	63 (11.9)	0.43 (0.29-0.64)	
12+	187 (14.6)	71 (9.5)	116 (21.9)	0.34 (0.27-0.47)	
**MDS comorbidity index**[2]					0.47
Low	780 (61)	466 (77.5)	314 (46.4)	1 (ref)	
Intermediate	414 (32.4)	238 (39.6)	176 (26)	0.91 (0.72 - 1.16)	
High	84 (6.6)	45 (7.5)	39 (5.8)	0.78 (0.49 - 1.22)	
**Dyspnoea level**[2]					0.44
None	1055 (82.6)	629 (104.7)	426 (62.9)	1 (ref)	
Moderate	141 (11)	76 (12.6)	65 (9.6)	0.79 (0.56 - 1.13)	
Slight	66 (5.2)	37 (6.2)	29 (4.3)	0.86 (0.52 - 1.43)	
Rest	13 (1)	6 (1)	7 (1)	0.58 (0.19 - 1.74)	
**Karnofsky Status**[2]					
Unable to care for self	29 (2.3)	8 (1.1)	21 (4)	0.25 (0.11 - 0.56)	0.0004
Unable to work	277 (21.7)	150 (20)	127 (24)	0.76 (0.58 - 1)	
Able to work/normal activity	966 (75.6)	587 (78.4)	379 (71.6)	1 (ref)	
**Country**[1]					<0.001
Austria	70 (5.5)	36 (6)	34 (5)	0.32 (0.18 - 0.55)	
Croatia	8 (0.6)	1 (0.2)	7 (1)	0.04 (0.01 - 0.35)	
Czech Republic	94 (7.4)	37 (6.2)	57 (8.4)	0.19 (0.12 - 0.32)	
Denmark	44 (3.4)	33 (5.5)	11 (1.6)	0.9 (0.42 - 1.89)	
France	235 (18.4)	181 (30.1)	54 (8)	1 (ref)	
Germany	36 (2.8)	13 (2.2)	23 (3.4)	0.17 (0.08 - 0.36)	
Greece	150 (11.7)	112 (18.6)	38 (5.6)	0.88 (0.55 - 1.42)	
Israel	94 (7.4)	62 (10.3)	32 (4.7)	0.58 (0.34 - 0.98)	
Italy	42 (3.3)	32 (5.3)	10 (1.5)	0.95 (0.44 - 2.07)	
Netherlands	42 (3.3)	21 (3.5)	21 (3.1)	0.3 (0.15 - 0.59)	
Poland	40 (3.1)	15 (2.5)	25 (3.7)	0.18 (0.09 - 0.36)	
Portugal	27 (2.1)	17 (2.8)	10 (1.5)	0.51 (0.22 - 1.17)	
Romania	32 (2.5)	23 (3.8)	9 (1.3)	0.76 (0.33 - 1.75)	
Republic of Serbia	22 (1.7)	-	22 (3.2)	-	
Spain	62 (4.9)	40 (6.7)	22 (3.2)	0.54 (0.3 - 0.99)	
Sweden	46 (3.6)	32 (5.3)	14 (2.1)	0.68 (0.34 - 1.37)	
UK	234 (18.3)	94 (15.6)	140 (20.7)	0.2 (0.13 - 0.3)	

ESA, erythropoiesis-stimulating agent; 95% CI, confidence intervals, MDS, myelodysplastic syndrome; RA, refractory anaemia; RARS, refractory anaemia with ring sideroblasts; RCMD, refractory cytopenia with multilineage dysplasia; RCMD-RS, refractory cytopenia with multilineage dysplasia and ring sideroblasts; RAEB, refractory anaemia with excess blasts; MDS-U, myelodysplastic syndrome, unclassifiable; IPSS-R, revised International Prognostic Scoring System.
[1] Diagnosis, [2] visit the eligibility criteria was reached.

Table S2: Baseline characteristics of eligible patients (haemoglobin <100 g/L) by exposure to erythropoietin-stimulating agents (ESA) and the association between each factor and the probability of ESA exposure in a multivariable logistic regression model

	Total N (%)	ESA Exposed N (%) Yes	No	Adjusted Odds Ratio (95% CI)	p-value
**Total**	1278 (100)	749 (58.6)	529 (41.4)		
**Age at Diagnosis (years)**					
<60	117 (9.2)	55 (7.3)	62 (11.7)	0.50 (0.32 - 0.77)	0.001
60-74	533 (41.7)	314 (41.9)	219 (41.4)	0.88 (0.68 - 1.14)	
75+	628 (49.1)	380 (50.7)	248 (46.9)	1 (ref)	
**Sex**					
Male	774 (60.6)	449 (74.7)	325 (48)	1 (ref)	0.59
Female	504 (39.4)	300 (49.9)	204 (30.1)	1.00 (0.78 - 1.28)	
**MDS diagnosis**[1]					
RA	203 (15.9)	141 (23.5)	62 (9.2)	2.03 (1.40 - 2.94)	0.001
RARS	215 (16.8)	154 (25.6)	61 (9)	2.27 (1.58 - 3.27)	
RCMD	499 (39)	260 (43.3)	239 (35.3)	1 (ref)	
RCMD-RS	84 (6.6)	56 (9.3)	28 (4.1)	1.65 (1.00 - 2.74)	
RAEB-1 or RAEB-2	153 (12)	70 (11.6)	83 (12.3)	0.61 (0.30 - 1.26)	
MDS-U	37 (2.9)	17 (2.8)	20 (3)	0.90 (0.44 - 1.85)	
5q-syndrome	87 (6.8)	51 (8.5)	36 (5.3)	1.16 (0.70 - 1.91)	
**IPSS-R risk category**[2]					
Very low	46 (3.6)	29 (4.8)	17 (2.5)	0.80 (0.42 - 1.55)	0.001
Low	665 (52)	436 (72.5)	229 (33.8)	1 (ref)	
Intermediate	308 (24.1)	172 (28.6)	136 (20.1)	0.71 (0.51 - 0.97)	
High/very high	107 (8.4)	41 (6.8)	66 (9.7)	0.38 (0.23 - 0.63)	
Unknown	152 (11.9)	71 (11.8)	81 (12)	0.54 (0.37 - 0.79)	
**Bone marrow blasts**[1]					0.01
<5%	1114 (87.2)	668 (111.1)	446 (65.9)	1 (ref)	
5-10%	164 (12.8)	81 (13.5)	83 (12.3)	0.75 (0.52 - 1.10)	
**Haemoglobin (g/L)**					0.20
80-100	1057 (82.7)	628 (83.8)	429 (81.1)	1 (ref)	
<80	221 (17.3)	121 (16.2)	100 (18.9)	0.81 (0.59 - 1.11)	
**Serum erythropoietin iU/L**					<0.0001
<100	95 (18.0)	222 (29.6)	95 (18.0)	1 (ref)	
100-500	75 (14.2)	136 (18.2)	75 (14.2)	0.78 (0.54-1.12)	
>500	44 (8.3)	31 (4.1)	44 (8.3)	0.30 (0.18-0.51)	
Not measured	315 (59.5)	360 (48.1)	315 (59.5)	-	
**Transfusions prior to reaching the criteria**					
No	675 (52.8)	360 (48.1)	284 (41.9)	1 (ref)	0.04
Yes	550 (43)	305 (50.7)	245 (36.2)	0.79 (0.61 - 1.01)	

	Total N (%)	ESA Exposed N (%)		Adjusted Odds Ratio (95% CI)	p-value
		Yes	No		
**Time from diagnosis to reaching the criteria (months)**					
<6	979 (76.6)	629 (84.0)	350 (66.2)	1 (ref)	<0.0001
6-12	112 (8.8)	49 (6.5)	63 (11.9)	0.41 (0.27 - 0.62)	
12+	187 (14.6)	71 (9.5)	116 (21.9)	0.30 (0.21 - 0.42)	
**MDS comorbidity index[2]**					0.47
Low	780 (61)	466 (77.5)	314 (46.4)	1 (ref)	
Intermediate	414 (32.4)	238 (39.6)	176 (26)	0.85 (0.65 - 1.11)	
High	84 (6.6)	45 (7.5)	39 (5.8)	0.68 (0.41 - 1.13)	
**Dyspnoea level[2]**					0.44
None	1055 (82.6)	629 (104.7)	426 (62.9)	1 (ref)	
Moderate	141 (11)	76 (12.6)	65 (9.6)	0.91 (0.62 - 1.33)	
Slight	66 (5.2)	37 (6.2)	29 (4.3)	1.12 (0.65 - 1.92)	
Rest	13 (1)	6 (1)	7 (1)	0.70 (0.21 - 2.40)	
**Karnofsky Status[2]**					
Unable to care for self	29 (2.3)	8 (1.1)	21 (4)	0.25 (0.10 - 0.60)	0.0004
Unable to work	277 (21.7)	150 (20)	127 (24)	0.77 (0.57 - 1.03)	
Able to work/normal activity	966 (75.6)	587 (78.4)	379 (71.6)	1 (ref)	
**Country[1]**					0.001
Austria	70 (5.5)	36 (6)	34 (5)	0.34 (0.19 - 0.62)	
Croatia	8 (0.6)	1 (0.2)	7 (1)	0.03 (0.00 - 0.22)	
Czech Republic	94 (7.4)	37 (6.2)	57 (8.4)	0.20 (0.11 - 0.34)	
Denmark	44 (3.4)	33 (5.5)	11 (1.6)	0.91 (0.41 - 2.01)	
France	235 (18.4)	181 (30.1)	54 (8)	1 (ref)	
Germany	36 (2.8)	13 (2.2)	23 (3.4)	0.18 (0.08 - 0.39)	
Greece	150 (11.7)	112 (18.6)	38 (5.6)	0.77 (0.47 - 1.28)	
Israel	94 (7.4)	62 (10.3)	32 (4.7)	0.66 (0.38 - 1.16)	
Italy	42 (3.3)	32 (5.3)	10 (1.5)	0.91 (0.41 - 2.03)	
Netherlands	42 (3.3)	21 (3.5)	21 (3.1)	0.35 (0.17 - 0.72)	
Poland	40 (3.1)	15 (2.5)	25 (3.7)	0.19 (0.09 - 0.41)	
Portugal	27 (2.1)	17 (2.8)	10 (1.5)	0.45 (0.19 - 1.08)	
Romania	32 (2.5)	23 (3.8)	9 (1.3)	0.70 (0.29 - 1.69)	
Republic of Serbia	22 (1.7)	-	22 (3.2)	-	
Spain	62 (4.9)	40 (6.7)	22 (3.2)	0.58 (0.31 - 1.09)	
Sweden	46 (3.6)	32 (5.3)	14 (2.1)	0.88 (0.42 - 1.86)	
UK	234 (18.3)	94 (15.6)	140 (20.7)	0.22 (0.14 - 0.34)	

ESA, erythropoiesis-stimulating agent; 95% CI, confidence intervals, MDS, myelodysplastic syndrome; RA, refractory anaemia; RARS, refractory anaemia with ring sideroblasts; RCMD, refractory cytopenia with multilineage dysplasia; RCMD-RS, refractory cytopenia with multilineage dysplasia and ring sideroblasts; RAEB, refractory anaemia with excess blasts; MDS-U, myelodysplastic syndrome, unclassifiable; IPSS-R, revised International Prognostic Scoring System.
[1] Diagnosis, [2] visit the eligibility criteria was reached.

Figure S3: Distribution of propensity score across exposed and unexposed groups

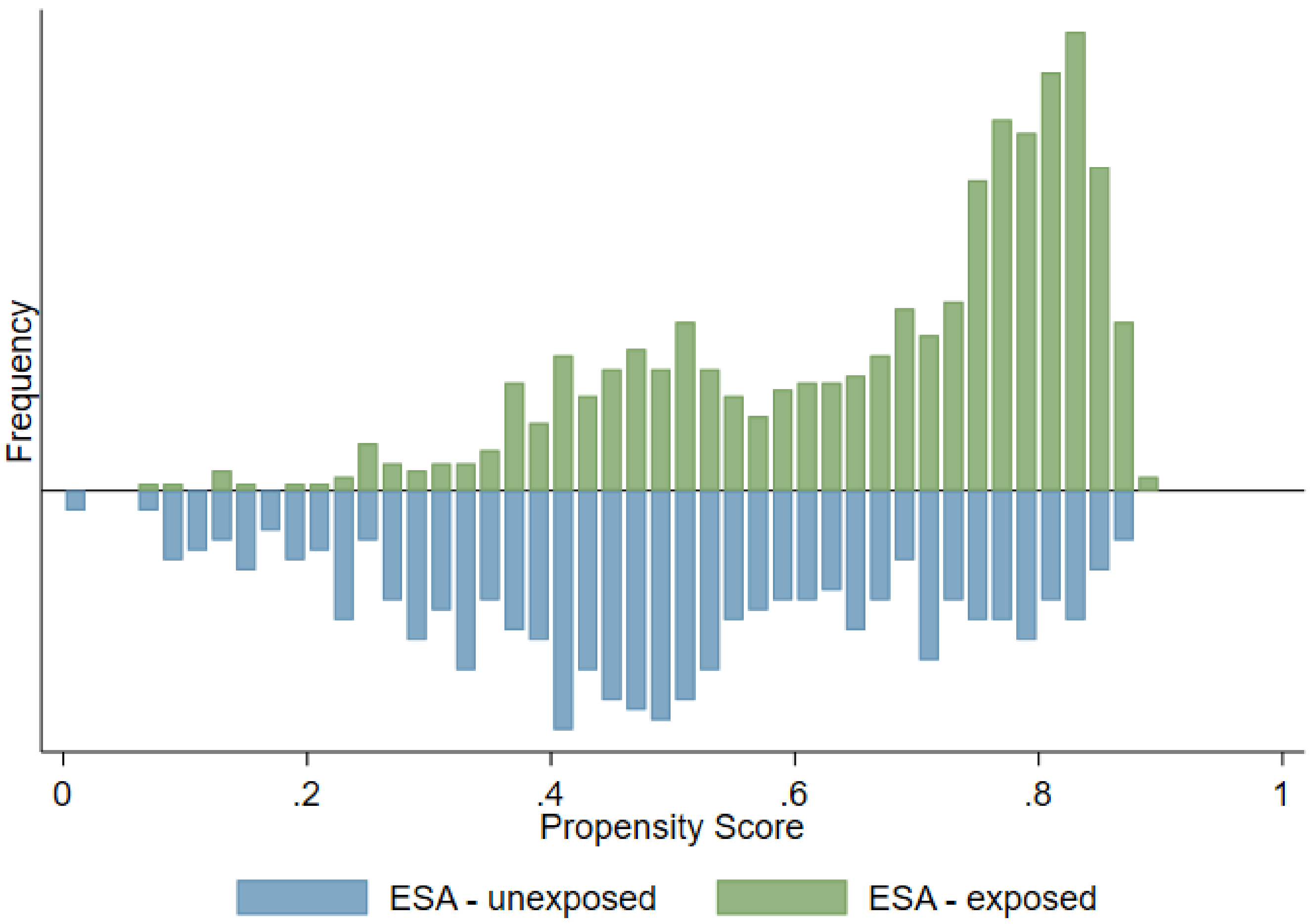

Table S3: Baseline characteristics of patients included in the propensity analysis by exposure to erythropoietin-stimulating agents (ESA) and the association between each factor and the probability of ESA exposure in a multivariable logistic regression model

	Total N (%)	ESA Exposed N (%)		Odds Ratio (95% CI)	p-value
		Yes	No		
**Total**	1170 (100.0)	744 (100.0)	426 (100.0)		
**Age at Diagnosis (years)**					0.10
<60	102 (8.7)	55 (7.4)	47 (11.0)	0.55 (0.35 - 0.86)	
60-74	488 (41.7)	312 (41.9)	176 (41.3)	0.93 (0.71 - 1.22)	
75+	580 (49.6)	377 (50.7)	203 (47.7)	1 (ref)	
**Sex**					0.61
Male	711 (60.8)	448 (60.2)	263 (61.7)	1 (ref)	
Female	459 (39.2)	296 (39.8)	163 (38.3)	0.96 (0.74 - 1.25)	
**MDS diagnosis**[1]					<0.0001
RA	193 (16.5)	140 (18.8)	53 (12.4)	1.95 (1.33 - 2.86)	
RARS	205 (17.5)	151 (20.3)	54 (12.7)	2.11 (1.45 - 3.08)	
RCMD	451 (38.5)	259 (34.8)	192 (45.1)	1 (ref)	
RCMD-RS	78 (6.7)	56 (7.5)	22 (5.2)	1.82 (1.05 - 3.13)	
RAEB-1 or RAEB-2	127 (10.9)	70 (9.4)	57 (13.4)	0.82 (0.38 - 1.80)	
MDS-U	34 (2.9)	17 (2.3)	17 (4.0)	0.82 (0.40 - 1.72)	
5q-syndrome	82 (7.0)	51 (6.9)	31 (7.3)	1.12 (0.66 - 1.88)	
**IPSS-R risk category**[2]					<0.0001
Very low	45 (3.8)	29 (3.9)	16 (3.8)	0.76 (0.39 - 1.48)	
Low	626 (53.5)	433 (58.2)	193 (45.3)	1 (ref)	
Intermediate	284 (24.3)	171 (23.0)	113 (26.5)	0.70 (0.50 - 0.98)	
High/very high	80 (6.8)	41 (5.5)	39 (9.2)	0.49 (0.29 - 0.85)	
Unknown	135 (11.5)	70 (9.4)	65 (15.3)	0.53 (0.36 - 0.79)	
**Bone marrow blasts**[1]					
<5%	1030 (88.0)	663 (89.1)	367 (86.2)	1 (ref)	0.17
5-10%	140 (12.0)	81 (10.9)	59 (13.8)	0.86 (0.58 - 1.28)	
**Haemoglobin (g/L)**					0.91
80-100	974 (83.2)	623 (83.7)	351 (82.4)	1 (ref)	
<80	196 (16.8)	121 (16.3)	75 (17.6)	0.88 (0.63 - 1.23)	
**Transfusions prior to reaching the criteria**					0.14
No	673 (57.5)	440 (59.1)	233 (54.7)	1 (ref)	
Yes	497 (42.5)	304 (40.9)	193 (45.3)	0.78 (0.60 - 1.02)	
**Time from diagnosis to reaching the criteria (months)**					0.001
<6	917 (78.4)	624 (83.9)	293 (68.8)	`1 (ref)	
6-12	100 (8.5)	49 (6.6)	51 (12.0)	0.43 (0.28 - 0.66)	
12+	153 (13.1)	71 (9.5)	82 (19.2)	0.35 (0.24 - 0.51)	

	Total N (%)	ESA Exposed N (%)		Odds Ratio (95% CI)	p-value
		Yes	No		
**MDS comorbidity index[2]**					0.82
Low	976 (83.4)	626 (84.1)	350 (82.2)	1 (ref)	
Intermediate	127 (10.9)	75 (10.1)	52 (12.2)	0.87 (0.65 - 1.15)	
High	57 (4.9)	37 (5.0)	20 (4.7)	0.71 (0.42 - 1.21)	
**Dyspnoea level[2]**					
None	976 (83.4)	626 (84.1)	350 (82.2)	1 (ref)	0.72
Moderate	127 (10.9)	75 (10.1)	52 (12.2)	0.91 (0.61 - 1.36)	
Slight	57 (4.9)	37 (5.0)	20 (4.7)	1.21 (0.67 - 2.19)	
Rest	10 (0.9)	6 (0.8)	4 (0.9)	0.72 (0.19 - 2.69)	
**Karnofsky Status[2]**					0.07
Unable to care for self	20 (1.7)	8 (1.1)	12 (2.8)	0.40 (0.15 - 1.03)	
Unable to work	243 (20.8)	149 (20.0)	94 (22.1)	0.85 (0.62 - 1.17)	
Able to work/normal activity	907 (77.5)	587 (78.9)	320 (75.1)	1 (ref)	
**Country[1]**					
Austria	61 (5.2)	35 (4.7)	26 (6.1)	0.39 (0.21 - 0.70)	<0.0001
Croatia	4 (0.3)	1 (0.1)	3 (0.7)	0.10 (0.01 - 0.94)	
Czech Republic	80 (6.8)	37 (5.0)	43 (10.1)	0.25 (0.14 - 0.42)	
Denmark	43 (3.7)	33 (4.4)	10 (2.3)	0.95 (0.44 - 2.05)	
France	229 (19.6)	178 (23.9)	51 (12.0)	1 (ref)	
Germany	32 (2.7)	13 (1.7)	19 (4.5)	0.20 (0.09 - 0.42)	
Greece	149 (12.7)	112 (15.1)	37 (8.7)	0.87 (0.53 - 1.41)	
Israel	91 (7.8)	62 (8.3)	29 (6.8)	0.61 (0.36 - 1.05)	
Italy	42 (3.6)	32 (4.3)	10 (2.3)	0.92 (0.42 - 1.99)	
Netherlands	36 (3.1)	20 (2.7)	16 (3.8)	0.36 (0.17 - 0.74)	
Poland	33 (2.8)	15 (2.0)	18 (4.2)	0.24 (0.11 - 0.51)	
Portugal	27 (2.3)	17 (2.3)	10 (2.3)	0.49 (0.21 - 1.13)	
Romania	32 (2.7)	23 (3.1)	9 (2.1)	0.73 (0.32 - 1.68)	
Republic of Serbia	59 (5.0)	40 (5.4)	19 (4.5)	0.60 (0.32 - 1.13)	
Spain	45 (3.8)	32 (4.3)	13 (3.1)	0.71 (0.34 - 1.44)	
Sweden	207 (17.7)	94 (12.6)	113 (26.5)	0.24 (0.16 - 0.36)	
UK	61 (5.2)	35 (4.7)	26 (6.1)		

ESA, erythropoiesis-stimulating agent; 95% CI, confidence intervals, MDS, myelodysplastic syndrome; RA, refractory anaemia; RARS, refractory anaemia with ring sideroblasts; RCMD, refractory cytopenia with multilineage dysplasia; RCMD-RS, refractory cytopenia with multilineage dysplasia and ring sideroblasts; RAEB, refractory anaemia with excess blasts; MDS-U, myelodysplastic syndrome, unclassifiable; IPSS-R, revised International Prognostic Scoring System.
[1] Diagnosis, [2] visit the eligibility criteria was reached.

Table S4: Results of χ^2 and t-tests comparing ESA exposed patients to patients not exposed to ESA with and without weighting by the propensity scores

	Total dataset						Propensity Model Dataset[3]					
	Unweighted			Weighted			Unweighted			**Weighted[5]**		
	Test Statistics[4]	df	p-value	Test Statistics[4]	df	p-value	Test Statistics[4]	df	p-value	**Test Statistics[4]**	**df**	**p-value**
Sex	0.29	1	0.59	0.07	1	0.79	0.26	1	0.61	**0.25**	**1**	**0.62**
MDS diagnosis[1]	49.44	6	<0.001	12.31	6	0.06	32.51	6	<0.001	**9.44**	**6**	**0.15**
IPSS-R risk category[2]	41.39	4	<0.001	0.48	4	0.97	22.86	4	<0.001	**0.81**	**4**	**0.94**
Bone marrow blasts[1]	6.50	1	0.01	0.01	1	0.93	2.22	1	0.14	**0.16**	**1**	**0.69**
Transfusions prior to reaching the eligibility criteria (months)	3.95	1	0.05	0.01	1	0.92	2.19	1	0.14	**0.00**	**1**	**0.99**
MDS comorbidity index[2]	1.50	2	0.47	0.03	2	0.98	0.40	2	0.82	**0.13**	**2**	**0.94**
Dyspnoea level[2]	3.49	4	0.48	0.14	3	0.99	1.34	3	0.72	**0.61**	**3**	**0.89**
Country	141.61	15	<0.001	0.25	15	1.00	94.43	15	<0.001	**2.30**	**15**	**1.00**
Karnofsky status[2]	15.55	2	<0.001	0.21	2	0.90	5.61	2	0.06	**0.19**	**2**	**0.91**
Age (years) [2]	2.35	1276	0.02	-0.39	1245	0.70	1.57	1168	0.12	**-0.68**	**1168**	**0.50**
Time from diagnosis to reaching the eligibility criteria (months) [2]	-6.38	1276	<0.001	0.51	1245	0.61	-4.78	1168	<0.001	**1.16**	**1168**	**0.25**
Haemoglobin[2]	0.90	1276	0.37	-0.19	1245	0.85	0.12	1168	0.90	**-0.33**	**1168**	**0.74**

[1]At diagnosis, [2]At time criteria reached. IPSS-R=revised International Prognostic Scoring System.
[3]Only includes patients from the common support region of the distribution of propensity scores.
[4]Categorical factors were compared using a χ^2 test; Non-categorical factors were compared using a t-test.
[5]Dataset used in the outcome analysis

Figure S4 Overall Survival by WHO Diagnostic Group

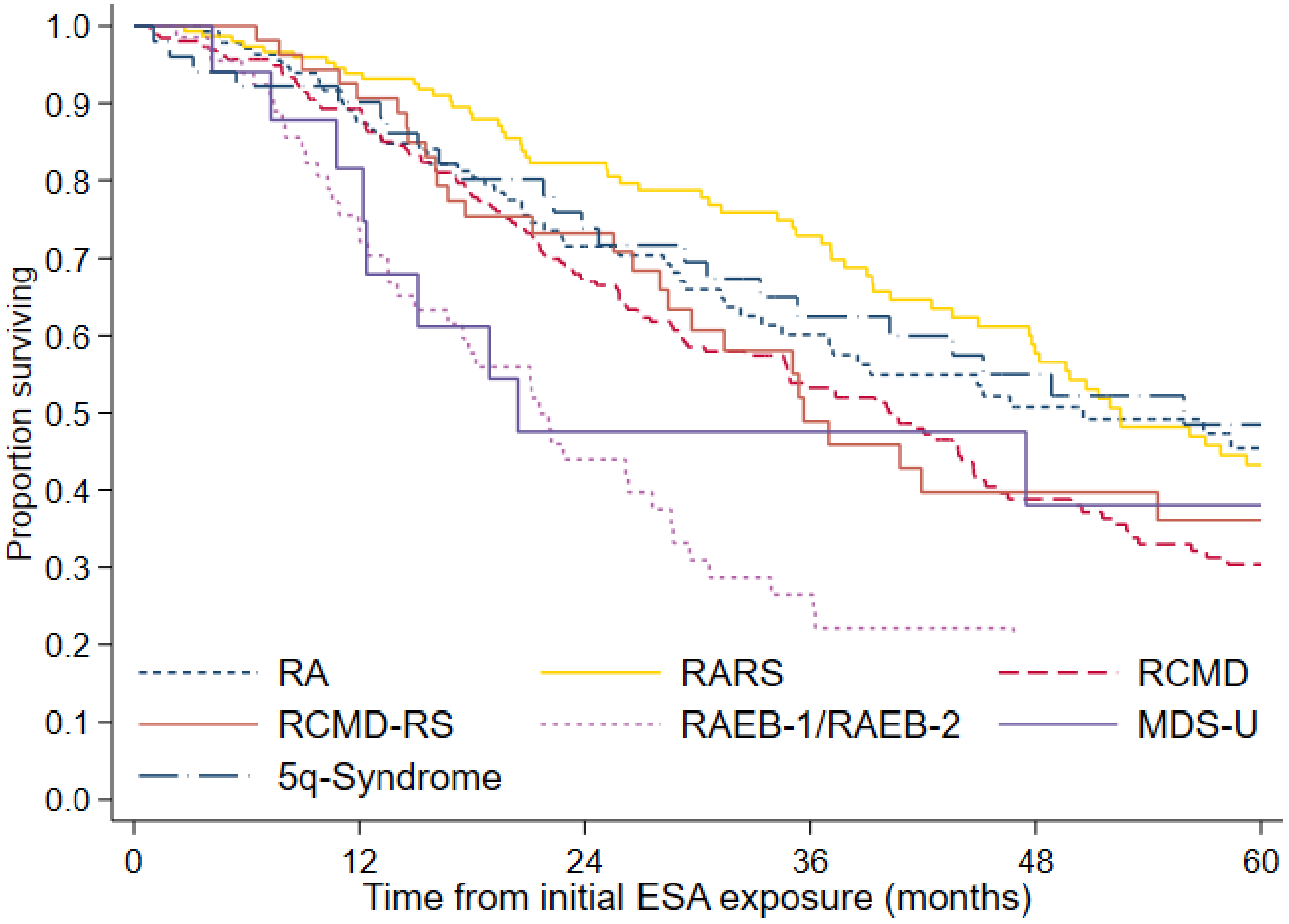

Figure S5 Overall Survival by the Revised International Prognostic Scoring System.

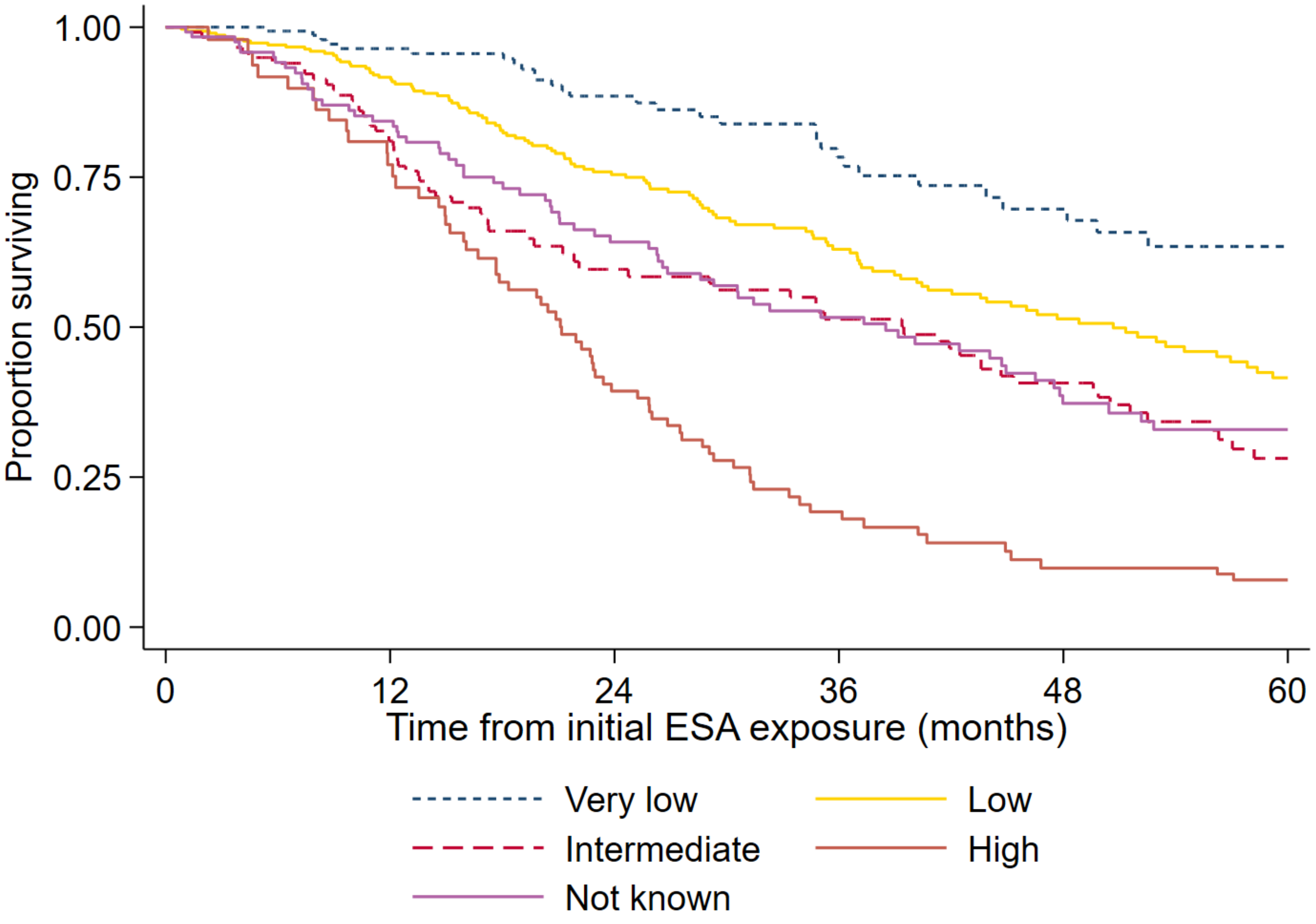

Table S5: EQ-5D questionnaires completed at visits 1 & 2 after meeting the criteria by baseline characteristics and included in the propensity analysis stratified by ESA exposure

	Total	ESA Exposed			
		Yes (n=744)		No (n=426)	
		HRQoL visits 1 & 2		HRQoL visits 1 & 2	
		No	Yes	No	Yes
**Total**	1170 (100.0)	347 (100.0)	397 (100.0)	242 (100.0)	184 (100.0)
**Sex**					
Female	459 (39.2)	136 (39.2)	160 (40.3)	91 (37.6)	72 (39.1)
Male	711 (60.8)	211 (60.8)	237 (59.7)	151 (62.4)	112 (60.9)
**Age at diagnosis**					
Mean (SD)	73.3 (9.8)	74.3 (9.3)	73.1 (9.2)	73.6 (10.3)	71.5 (10.9)
Median (min-max)	74.0 (21.0 - 97.0)	75.0 (41.0 - 97.0)	74.0 (36.0 - 95.0)	75.0 (34.0 - 92.0)	72.5 (21.0 - 93.0)
**Country**					
Austria	61 (5.2)	9 (2.6)	26 (6.5)	12 (5.0)	14 (7.6)
Croatia	4 (0.3)	1 (0.3)	0 (0.0)	3 (1.2)	0 (0.0)
Czech Republic	80 (6.8)	18 (5.2)	19 (4.8)	16 (6.6)	27 (14.7)
Denmark	43 (3.7)	15 (4.3)	18 (4.5)	4 (1.7)	6 (3.3)
France	229 (19.6)	104 (30.0)	74 (18.6)	41 (16.9)	10 (5.4)
Germany	32 (2.7)	13 (3.7)	0 (0.0)	19 (7.9)	0 (0.0)
Greece	149 (12.7)	42 (12.1)	70 (17.6)	19 (7.9)	18 (9.8)
Israel	91 (7.8)	36 (10.4)	26 (6.5)	26 (10.7)	3 (1.6)
Italy	42 (3.6)	27 (7.8)	5 (1.3)	10 (4.1)	0 (0.0)
Netherlands	36 (3.1)	9 (2.6)	11 (2.8)	10 (4.1)	6 (3.3)
Poland	33 (2.8)	7 (2.0)	8 (2.0)	12 (5.0)	6 (3.3)
Portugal	27 (2.3)	17 (4.9)	0 (0.0)	10 (4.1)	0 (0.0)
Romania	32 (2.7)	6 (1.7)	17 (4.3)	6 (2.5)	3 (1.6)
Spain	59 (5.0)	12 (3.5)	28 (7.1)	4 (1.7)	15 (8.2)
Sweden	45 (3.8)	6 (1.7)	26 (6.5)	4 (1.7)	9 (4.9)

	Total	ESA Exposed			
		Yes (n=744)		No (n=426)	
		HRQoL visits 1 & 2		HRQoL visits 1 & 2	
		No	Yes	No	Yes
United Kingdom	207 (17.7)	25 (7.2)	69 (17.4)	46 (19.0)	67 (36.4)
**IPSSR**					
Very low	45 (3.8)	14 (4.0)	15 (3.8)	8 (3.3)	8 (4.3)
Low	626 (53.5)	186 (53.6)	247 (62.2)	105 (43.4)	88 (47.8)
Intermediate	284 (24.3)	85 (24.5)	86 (21.7)	64 (26.4)	49 (26.6)
High	80 (6.8)	25 (7.2)	16 (4.0)	26 (10.7)	13 (7.1)
Not known	135 (11.5)	37 (10.7)	33 (8.3)	39 (16.1)	26 (14.1)
**Karnofsky performance status**					
≤40 (unable to care for self)	20 (1.7)	5 (1.4)	3 (0.8)	8 (3.3)	4 (2.2)
50-70 (unable to work)	243 (20.8)	70 (20.2)	79 (19.9)	57 (23.6)	37 (20.1)
80-100 (able to work and normal activity)	907 (77.5)	272 (78.4)	315 (79.3)	177 (73.1)	143 (77.7)
**Bone marrow blast count (%)**					
<5	1030 (88.0)	304 (87.6)	359 (90.4)	206 (85.1)	161 (87.5)
5-10	140 (12.0)	43 (12.4)	38 (9.6)	36 (14.9)	23 (12.5)
**MDS comorbidity index**					
Low risk	720 (61.5)	218 (62.8)	244 (61.5)	149 (61.6)	109 (59.2)
Intermediate risk	376 (32.1)	107 (30.8)	130 (32.7)	76 (31.4)	63 (34.2)
High risk	74 (6.3)	22 (6.3)	23 (5.8)	17 (7.0)	12 (6.5)
**Dyspnoea**					
None	976 (83.4)	301 (86.7)	325 (81.9)	202 (83.5)	148 (80.4)
Moderate	127 (10.9)	27 (7.8)	48 (12.1)	26 (10.7)	26 (14.1)
Slight	57 (4.9)	18 (5.2)	19 (4.8)	14 (5.8)	6 (3.3)
Rest	10 (0.9)	1 (0.3)	5 (1.3)	0 (0.0)	4 (2.2)

	Total	ESA Exposed			
		Yes (n=744)		No (n=426)	
		HRQoL visits 1 & 2		HRQoL visits 1 & 2	
		No	Yes	No	Yes
**Time from diagnosis to criteria (months)**					
Mean (SD)	5.5 (10.4)	4.4 (8.3)	4.4 (9.4)	8.2 (13.8)	6.3 (10.0)
Median (min-max)	1.9 (0.0 - 111.0)	1.7 (0.0 - 74.4)	1.6 (0.0 - 73.5)	2.2 (0.0 - 111.0)	2.4 (0.0 - 71.6)
**Haemoglobin level (g/L)**					
Mean (SD)	88 (10)	88 (10)	89 (90)	88 (10)	88 (10)
Median (min-max)	91 (42 - 100)	91 (42 - 100)	91 (49 - 100)	92 (44 - 100)	9.1 (56 - 10)
Median follow-up time (years)<br>95% Confidence Intervals	4.4 (4.1 - 4.8)	3.6 (3.3 - 4.2)	5.4 (4.7 - 5.9)	3.2 (2.7 - 4.1)	6.4 (4.6 - 8.1)
Median Survival (years)<br>95% Confidence Intervals	3.3 (3.0 - 3.7)	3.6 (2.8 - 4.2)	4.0 (3.5 - 4.7)	2.0 (1.5 - 2.4)	3.2 (2.7 - 3.8)

Table S6: Characteristics by Transfusion Status Prior to ESA exposure

	Total N (%)	Transfused Prior to starting ESA N (%)		Number of Units	
		No	Yes	≤4	>4
**Total**	749 (100.0)	396 (100.0)	353 (100.0)	146 (100.0)	207 (100.0)
**Age at Diagnosis (years)**					
<60	55 (7.3)	37 (9.3)	18 (5.1)	10 (6.8)	8 (3.9)
60-74	314 (41.9)	156 (39.4)	158 (44.8)	56 (38.4)	102 (49.3)
75+	380 (50.7)	203 (51.3)	177 (50.1)	80 (54.8)	97 (46.9)
**Sex**					
Male	449 (59.9)	233 (58.8)	216 (61.2)	89 (61.0)	127 (61.4)
Female	300 (40.1)	163 (41.2)	137 (38.8)	57 (39.0)	80 (38.6)
**MDS diagnosis**[1]					
RA	141 (18.8)	69 (17.4)	72 (20.4)	36 (24.7)	36 (17.4)
RARS	154 (20.6)	91 (23.0)	63 (17.8)	21 (14.4)	42 (20.3)
RCMD	260 (34.7)	139 (35.1)	121 (34.3)	52 (35.6)	69 (33.3)
RCMD-RS	56 (7.5)	30 (7.6)	26 (7.4)	7 (4.8)	19 (9.2)
RAEB-1 or RAEB-2	70 (9.3)	32 (8.1)	38 (10.8)	19 (13.0)	19 (9.2)
MDS-U	17 (2.3)	9 (2.3)	8 (2.3)	4 (2.7)	4 (1.9)
5q-syndrome					
**IPSS-R risk category**[2]	37 (4.9)	22 (5.6)	15 (4.2)	10 (6.8)	5 (2.4)
Very low	427 (57.0)	240 (60.6)	187 (53.0)	81 (55.5)	106 (51.2)
Low	164 (21.9)	74 (18.7)	90 (25.5)	33 (22.6)	57 (27.5)
Intermediate	43 (5.7)	21 (5.3)	22 (6.2)	10 (6.8)	12 (5.8)
High/very high	78 (10.4)	39 (9.8)	39 (11.0)	12 (8.2)	27 (13.0)
Unknown	37 (4.9)	22 (5.6)	15 (4.2)	10 (6.8)	5 (2.4)
**Bone marrow blasts**[1]					
<5%	669 (89.3)	358 (90.4)	311 (88.1)	127 (87.0)	184 (88.9)
5-10%	80 (10.7)	38 (9.6)	42 (11.9)	19 (13.0)	23 (11.1)
**Haemoglobin (g/L)** [2]					
>=100	23 (3.1)	14 (3.5)	9 (2.5)	4 (2.7)	5 (2.4)
80-100	596 (79.6)	341 (86.1)	255 (72.2)	112 (76.7)	143 (69.1)
<80	130 (17.4)	41 (10.4)	89 (25.2)	30 (20.5)	59 (28.5)
**MDS comorbidity index**[2]					
Low	477 (63.7)	267 (67.4)	210 (59.5)	82 (56.2)	128 (61.8)
Intermediate	232 (31.0)	104 (26.3)	128 (36.3)	58 (39.7)	70 (33.8)
High	40 (5.3)	25 (6.3)	15 (4.2)	6 (4.1)	9 (4.3)
**Dyspnoea level**[2]					
None	628 (84.0)	334 (84.6)	294 (83.3)	129 (88.4)	165 (79.7)
Moderate	77 (10.3)	38 (9.6)	39 (11.0)	11 (7.5)	28 (13.5)
Slight	38 (5.1)	21 (5.3)	17 (4.8)	6 (4.1)	11 (5.3)
Rest	5 (0.7)	2 (0.5)	3 (0.8)	0 (0.0)	3 (1.4)

	Total N (%)	Transfused Prior to starting ESA N (%)		Number of Units	
		No	Yes	≤4	>4
**Karnofsky Status[2]**					
Unable to care for self	8 (1.1)	3 (0.8)	5 (1.4)	3 (2.1)	2 (1.0)
Unable to work	149 (20.0)	59 (15.1)	90 (25.6)	38 (26.2)	52 (25.1)
Able to work/normal activity	587 (78.9)	330 (84.2)	257 (73.0)	104 (71.7)	153 (73.9)

[1] Diagnosis, [2] visit prior to ESA start

Table S7: Exposed group at visit 1 after reaching the criteria for patients that completed EQ-5D questionnaires at visits 1 & 2 by baseline characteristics and included in the propensity analysis

	Total	Exposed group at visit 1 after reaching the criteria			
		No ESA, No RBCT	ESA, No RBCT	ESA, RBCT	No ESA, RBCT
**Total**	581 (100.0)	244 (100.0)	64 (100.0)	68 (100.0)	205 (100.0)
**Sex**					
Female	232 (39.9)	104 (42.6)	27 (42.2)	26 (38.2)	75 (36.6)
Male	349 (60.1)	140 (57.4)	37 (57.8)	42 (61.8)	130 (63.4)
**Age at diagnosis**					
Mean (SD)	72.6 (9.8)	72.2 (9.7)	74.6 (8.7)	73.1 (9.5)	72.3 (10.3)
Median (min-max)	73.0 (21.0 - 95.0)	73.0 (36.0 - 93.0)	75.0 (46.0 - 90.0)	75.0 (46.0 - 89.0)	72.0 (21.0 - 95.0)
**Country**					
Austria	40 (6.9)	22 (9.0)	4 (6.2)	3 (4.4)	11 (5.4)
Czech Republic	46 (7.9)	22 (9.0)	0 (0.0)	3 (4.4)	21 (10.2)
Denmark	24 (4.1)	9 (3.7)	2 (3.1)	9 (13.2)	4 (2.0)
France	84 (14.5)	43 (17.6)	14 (21.9)	10 (14.7)	17 (8.3)
Greece	88 (15.1)	20 (8.2)	15 (23.4)	17 (25.0)	36 (17.6)
Israel	29 (5.0)	6 (2.5)	5 (7.8)	7 (10.3)	11 (5.4)
Italy	5 (0.9)	0 (0.0)	1 (1.6)	1 (1.5)	3 (1.5)
Netherlands	17 (2.9)	5 (2.0)	3 (4.7)	1 (1.5)	8 (3.9)
Poland	14 (2.4)	5 (2.0)	0 (0.0)	1 (1.5)	8 (3.9)
Romania	20 (3.4)	13 (5.3)	1 (1.6)	0 (0.0)	6 (2.9)
Spain	43 (7.4)	22 (9.0)	2 (3.1)	6 (8.8)	13 (6.3)
Sweden	35 (6.0)	12 (4.9)	3 (4.7)	4 (5.9)	16 (7.8)
United Kingdom	136 (23.4)	65 (26.6)	14 (21.9)	6 (8.8)	51 (24.9)
**IPSSR**					
Very low	23 (4.0)	15 (6.1)	3 (4.7)	1 (1.5)	4 (2.0)

	Total	Exposed group at visit 1 after reaching the criteria			
		No ESA, No RBCT	ESA, No RBCT	ESA, RBCT	No ESA, RBCT
Low	335 (57.7)	147 (60.2)	39 (60.9)	34 (50.0)	115 (56.1)
Intermediate	135 (23.2)	42 (17.2)	15 (23.4)	24 (35.3)	54 (26.3)
High	29 (5.0)	15 (6.1)	1 (1.6)	3 (4.4)	10 (4.9)
Not known	59 (10.2)	25 (10.2)	6 (9.4)	6 (8.8)	22 (10.7)
**Karnofsky performance status**					
≤40 (unable to care for self)	7 (1.2)	2 (0.8)	0 (0.0)	1 (1.5)	4 (2.0)
50-70 (unable to work)	116 (20.0)	41 (16.8)	13 (20.3)	15 (22.1)	47 (22.9)
80-100 (able to work and normal activity)	458 (78.8)	201 (82.4)	51 (79.7)	52 (76.5)	154 (75.1)
**Bone marrow blast count (%)**					
<5	520 (89.5)	222 (91.0)	56 (87.5)	58 (85.3)	184 (89.8)
5-10	61 (10.5)	22 (9.0)	8 (12.5)	10 (14.7)	21 (10.2)
**MDS comorbidity index**					
Low risk	353 (60.8)	161 (66.0)	38 (59.4)	42 (61.8)	112 (54.6)
Intermediate risk	193 (33.2)	69 (28.3)	19 (29.7)	24 (35.3)	81 (39.5)
High risk	35 (6.0)	14 (5.7)	7 (10.9)	2 (2.9)	12 (5.9)
**Dyspnoea**					
None	473 (81.4)	207 (84.8)	52 (81.2)	57 (83.8)	157 (76.6)
Moderate	74 (12.7)	28 (11.5)	7 (10.9)	9 (13.2)	30 (14.6)
Slight	25 (4.3)	6 (2.5)	5 (7.8)	2 (2.9)	12 (5.9)
Rest	9 (1.5)	3 (1.2)	0 (0.0)	0 (0.0)	6 (2.9)
**Time from diagnosis to criteria (months)**					
Mean (SD)	5.0 (9.6)	7.1 (12.2)	2.3 (2.2)	2.7 (4.3)	4.1 (8.3)
Median (min-max)	1.8 (0.0 - 73.5)	1.9 (0.0 - 73.5)	1.9 (0.2 - 13.0)	2.1 (0.2 - 31.9)	1.6 (0.0 - 70.1)
**Haemoglobin level (g/L)**					
Mean (SD)	89 (10)	92 (80)	91 (70)	84 (10)	85 (10)

	Total	Exposed group at visit 1 after reaching the criteria			
		**No ESA, No RBCT**	**ESA, No RBCT**	**ESA, RBCT**	**No ESA, RBCT**
Median (min-max)	9.1 (4.9 - 10.0)	9.5 (4.9 - 10.0)	9.3 (6.5 - 9.9)	8.5 (4.9 - 9.9)	8.7 (5.0 - 10.0)
**Number of EQ-5D completed in visits 1-6**					
2 (visit 1 & 2 after reaching the criteria only)	157 (27.0)	64 (26.2)	13 (20.3)	17 (25.0)	63 (30.7)
>2	424 (73.0)	180 (73.8)	51 (79.7)	51 (75.0)	142 (69.3)
Mean (sd)	5.0 (3.5)	5.4 (3.9)	4.7 (3.4)	4.1 (2.3)	4.9 (3.4)
**Number of visits after reaching criteria**					
2	101 (17.4)	43 (17.6)	9 (14.1)	9 (13.2)	40 (19.5)
>2	480 (82.6)	201 (82.4)	55 (85.9)	59 (86.8)	165 (80.5)
Mean (sd)	6.4 (4.3)	6.8 (4.4)	6.3 (4.2)	5.1 (3.1)	6.2 (4.3)
Median follow-up time (years) 95% Confidence Intervals	4.9 (4.5-5.6)	5.3 (4.3-5.9)	4.7 (2.8-5.0)	4.1 (2.9-4.9)	6.1 (4.6-7.7)
Median survival time (years) 95% Confidence Intervals	3.7 (3.3 - 4.0)	4.7 (3.9 - 5.8)	3.7 (3.1 - 5.8)	2.5 (2.0 - 3.3)	3.2 (2.7 - 3.9)

Table S8: Reimbursement practice - personal information

	Is ESA for MDS patients with anemia reimbursed / possible to give?<br>a. No<br>b. Yes, but only for transfusion-dependent patients<br>c. Yes, for any patient with symptomatic anemia	
Country	Yes / No<br>- restrictions	Explanation
Austria	Yes	for any patient with symptomatic anemia
Belgium	Yes	only epo-alpha is reimbursed for MDS patients with symptomatic anemia (defined as Hb =< 10g/L), IPSS low and intermediate-1, EPO < 200 U/L, and primary MDS
Bulgaria	**No**	ESAs are not reimbursed for MDS
Croatia	Yes - **TD**	ESA is possible to give as reimbursed drug for transfusion-dependent and symptomatic anemia patients
Czech Republic	Yes	for MDS patients, there are no general limits<br>*Comment*: ESA are mostly used in patients with sEPO below 200 IU. This is also a recommendation in our national guidelines.
Denmark	Yes	for any patient with symptomatic anemia
France	Yes	for any patient with symptomatic anemia
Germany	Yes - **TD**	only EPO-Alpha is reimbursed for transfusion-dependent patients with IPSS low/intermediate-1 and only if endo EPO Level is <200
Greece	Yes	in symptomatic patients with an Hb < 10gr/dL and low/intermediate-1 risk disease who have serum EPO levels < 200mU/ml.<br>*Comment*: the Greek Authorities reimburse ESA to all symptomatic patients for a maximum period of 16 weeks before a final evaluation of response is made. For patients, who respond to treatment, according to their physician evaluation, treatment can be continued as long as they merit benefit from this treatment
Israel	Yes - **TD**	the public system (covering 99%) of the population fully covers ESA for:<br>- MDS patients with Hb < 10 + transfusion dependence - all<br>- MDS patients with Hb <10, still not transfusion dependent, if they suffer from cardiovascular comorbidity<br>- All patients on dialysis - all<br>- Patients with serum creatinine > 2 , most of them<br>Others (10-20%) who have additional private insurance might receive for additional indications (any anemia below Hb 11)
Italy	Yes	for patients with MDS low or intermediate-1 according to IPSS, symptomatic anemia (Hb $\leq$ 10 d/dL) and sEPO < 200 U/L
Netherlands	Yes	for MDS patients with symptomatic anemia
Poland	Yes	for MDS patients with symptomatic anemia.<br>*Comment*: The most frequently used ESA is Darbepoetin
Portugal	Yes	all formulations of ESA are reimbursed for symptomatic anemia regardless of transfusion requirement.
Romania	Yes	for MDS patients with symptomatic anemia and EPO < 500 U/L.
Serbia	**No**	ESA is not reimbursed by obligatory national health fund. Some patients got it from other funds
Slovenia	Yes	for any patient with symptomatic anemia
Spain	Yes	for any patient with symptomatic anemia (Hb < 10.5 g/L, EPO < 500U/L)
Sweden	Yes	for any patient with symptomatic anemia
Switzerland	Yes	Yes for MDS patients with symptomatic anaemia and after request for reimbursement.<br>*Comment*: Darbeopoietin is on the reimbursement list based on the phase 3 trial, the others are not and sometimes more complicated to receive, even though they are less expensive.
United Kingdom	Yes	within the SPC for Eprex, namely symptomatic anaemia with Hb < or equal to 100 g/l irrespective of transfusion status
USA	Yes	for any MDS patient with a Hb <10.<br>*Comment*: The NCCN guidelines recognizes Hb 10 and serum EPO <= 500 (actually in practice < 300) with adequate iron stores as an important cutpoint but recommends ESA use for those patients whose anemia is symptomatic.

Yes	Yes, with restrictions	No
**17**	**3**	**2**

Table S9: List of EUMDS participants

Country	**Participating centers** (City, Name organization, *[local investigator(s)]*)
Austria	Innsbruck, Medical University of Innsbruck *[**R. Stauder**]*
	Wels, Klinikum Wels-Grieskirchen *[S. Burgstaller / J. Thaler]*
	Lienz, Bezirkskrankenhaus *[A. Walder]*
	Vienna, Hanusch Krankenhaus *[M. Pfeilstöcker / A. Schoenmetzler-Makrai]*
Croatia	Zagreb, Clinical Hospital Merkur *[**I. Mandac**]*
Czech Republic	Prague, General University Hospital, Institute of Hematology and Blood Transfusion *[**J. Cermak** / D. Mikulenková]*
	Prague, Motol University Hospital *[I. Hochova]*
	Brno, University Hospital Brno-Bohunice *[M. Krejci]*
	Prague, General University Hospital, 1st Clinic of Internal Medicine *[A. Jonasova]*
	Hradec Kralove, Charles University Faculty of Medicine *[J. Voglova]*
	Olomouc, University Hospital *[P. Rohon]*
Denmark	Aarhus, University Hospital *[**M.S. Holm**]*
	Odense, Odense University Hospital *[H. Vestergaard]*
	Copenhagen, University Hospital: Rigshospitalet *[L. Kjeldsen / K.Grønbæk]*
	Herlev Ringvej, Herlev Hospital *[I.H. Dufva]*
	Aalborg, University Hospital *[P.D. Jensen]*
France	Bobigny, Hospital Avicenne *[P. Fenaux]*
	Nancy, CHU Nancy: Hospital Brabois (Vandoeuvre Les Nancy) *[M. D'Aveni]*
	Paris, Hôpital St. Louis *[**P. Fenaux** / R. Itzykson / L. Adès]*
	Perpignan, Centre Hospital Maréchal Joffre *[L. Sanhes]*
	Avignon, Centre Hospital *[B. Slama]*
	Toulouse, CHU Toulouse: Hospital Purpan, Toulouse *[O. Beyne-Rauzy]*
	Lyon, Hospital Edouard Herriot *[E. Wattel]*
	Cochin, Hospital Hôtel Dieu *[L. Willems]*
	Chalon sur Saone, Centre Hospital William Morey *[D. Klepping / B. Salles]*
	Tours, CHRU de Tours *[E. Gyan]*
	Caen, Centre Hospital Universitaire Clemenceau *[S. Cheze]*
	Limoges, CHU Limoges Hospital Dupuytren *[M. Chaury]*
	Le Kremlin Bicêtre, Hospital Bicêtre *[G. Tertian]*
	Strasbourg, CHU Hospital Hautepierre de Strasbourg *[S. Amé]*
	Nice, CHU de Nice: Hospital l'Archet *[L. Legros]*
	Antibes, Hospital Center D'antibes Juan-Les-Pins *[D. Re]*
	Boulogne sur Mer, Centre Hospital Boulogne-sur-Mer *[B. Choufi]*
	Grenoble, CHU Albert Michallon *[S. Courby]*
	Lille - St Vincent, Hospital St Vincent de Paul *[L. Pascal]*
	Rouen, CHU de Rouen: Hospital Charles-Nicolle *[A. Stamatoullas]*
	Clermont-Ferrand, Centre Hospital Universitaire *[B. de Renzis]*
	Fréjus, CHI Frejus Saint Raphael *[J. Gutnecht]*
	Ivry sur Seine, Hôpital Charles-Foix Ap-Hp *[V. Siguret]*
	Colombes, Louis-Mourier Hospital [D. De Prost]
	Paris, Hospital Saint-Antoine *[F. Isnard]*
	Pontoise, Centre Hospital René Dubos Pontoise *[R. Benramdane]*
Germany	Düsseldorf, Heinrich-Heine University Hospital *[**U. Germing**]*
	Dresden, University Hospital Carl Gustav Carus *[U. Platzbecker]*
	Ulm, University Hospital Ulm *[R. Schlenk]*
	Duisburg, HELIOS: St. Johannes Hospital in Hamborn *[C. Badrakan]*
	Freiburg, University Hospital Freiburg *[M. Lübbert]*
Greece	Patras, General University Hospital of Patras *[**A. Symeonidis** / A. Kourakli]*
	Alexandroupolis, Democritus University of Thrace *[I. Kotsianidis / C. Tsatalas]*
	Athens, General Hospital Laikon - Propaedeutic Medicine, University of Athens Medical School *[P. Panagiotidis]*
	Athens, Pammakaristos Hospital *[A. Kostourou]*
	Athens, General Hospital G. Gennimatas *[A. Galanopoulos / E. Michali]*
	Chania, General Hospital of Chania *[K. Palla]*
	Athens, Patission Prefectural General Hospital: Halkida *[Z. Kartasis]*
	Athens, General Hospital Attikon, University of Athens Medical School *[V. Pappa]*
	Thessaloniki, Hippokration - General Hospital of Thessaloniki *[E. Vlachaki]*
	Patras, St. Andreas General Hospital *[P. Zikos]*
	Pilea Chortiatis, General Hospital of Thessaloniki George Papanikolaou *[A. Anagnostopoulos]*

Country	**Participating centers** (City, Name organization, *[local investigator(s)]*)
	Ioannina, University Hospital of Ioannina *[V. Briasoulis / E. Hatzimichael]*
	Athens, General Hospital Laikon - Internal Medicine, University of Athens Medical School *[N. Viniou]*
	Piraeus, Metaxa Hospital *[M. Kotsopoulou / K. Megalakaki]*
	Athens, St. Savvas Oncology Hospital of Athens *[A. Pouli]*
	Serres, General Hospital of Serres *[M. Protopapa]*
	Athens, General Hospital Sotiria, University of Athens Medical School *[A. Katsigiannis / P. Roussou]*
	Athens, Hellenic 251 Air Force General Hospital *[E. Terpos]*
	Thessaloniki, Theageneio General Hospital *[P. Konstantinidou]*
	Larissa, University Hospital of Larissa *[G. Vassilopoulos]*
Israel	Tel Aviv, Tel Aviv Sourasky (Ichilov) Medical Centre *[**M. Mittelman**]*
	Rehovot, Kaplan Medical Center *[K. Filanovsky]*
	Nahariya, The Western Galilee Hospital *[A. Braester]*
	Beersheba, Soroka Medical Center *[E. Levy / U. Greenbaum]*
	Haifa, Rambam Medical Centre *[Y. Ofran]*
	Kfar Saba, Meir Medical Center *[I. Hellman / M. Ellis]*
	Afula, HaEmek Medical Center *[G. Stemer]*
	Be'er Ya'akov, Asaf-Harofe Medical Center *[U. Gotwin / O. Cohen / M. Koren]*
	Petah Tikva, Rabin Medical Center *[G. Itzhaki / O. Wolaj]*
	Ashkelon, Barzilai Medical Center *[A. Nemetz]*
	Jerusalem, Hadassah Medical Center *[N. Goldshmidt / S. Elias / R. Sabag]*
	Holon, Wolfson Medical Center *[A. Winder]*
	Netanya, Laniado Hospital *[S. Berdichevsky]*
	Haifa, Carmel Medical Center *[M. Price]*
	Haifa, Bnai Zion Medical Center *[S. Gino-Moor]*
	Tiberias, Baruch Padeh Medical Center Poriya *[S. Yeganeh / O. Katz]*
Italy	Pavia, University of Pavia Medical School, IRCCS San Matteo Hospital Foundation *[**L. Malcovati**]*
	Rome, University Cattolica del Sacro Cuore, Policlinico Gemelli *[L. Fianchi]*
Netherlands	Nijmegen, Radboudumc *[**S. Langemeijer** / M. Hoeks]*
	Den Bosch, Jeroen Bosch Hospital *[A. Herbers / H. Pruijt]*
	Ede, Gelderse Vallei Hospital *[G. Velders]*
	Arnhem, Rijnstate Hospital *[V. Matthijssen]*
	Uden, Bernhoven Hospital *[C. Lensen]*
	Amsterdam, VU University Medical Center *[A. vd Loosdrecht]*
	Doetinchem, Slingeland Hospital *[N. Aboosy / F. de Vries]*
	Veldhoven, Maxima Medical Center *[P. Kuijper]*
	Helmond, Elkerliek Hospital *[E. Jacobs]*
Poland	Warsaw, Medical University of Warsaw *[**K. Madry**]*
Portugal	Lisbon, Centro Hospitalar de Lisboa *[M. Câmara / **A. Almeida**]*
Romania	Bucharest, Fundeni Clinical Institute *[**A. Tatic**]*
	Bucharest, Coltea Clinical Hospital *[O. Stanca Ciocan]*
	Brasov, Districtual Hospital *[G. Vulkan]*
Serbia	Novi Sad, Clinical Center of Vojvodina *[**A. Savic**]*
Spain	Oviedo, Hospital Universitario Central de Asturias *[T. Bernal]*
	Valencia, Hospital Clinico Universitario de Valencia *[D. Tormo]*
	Valencia, Hospital Universitario La Fe *[**G. Sanz**]*
	Lleida, Instituto de Investigación Biomédica *[V. Betés]*
	Barcelona, Hospital del Mar *[C. Pedro]*
	Valencia, Hospital Dr. Peset *[R. Andreu Lapiedra]*
	Salamaca, Hospital Universitario de Salamanca *[M. Diez Campelo]*
	Barcelona, Hospital Universitari Germans Trias i Pujol *[B. Xicoy]*
	Murcia, Hospital Universitario Meseguer *[M. Lozano / M. Martínez]*
	Cádiz, Hospital Del Sas, Jerez De La Frontera *[P. Leiva]*
	Cádiz, Hospital Universitario Puerta del Mar *[J. Muñoz]*
	Madrid, Hospital Clinico Universitario San Carlos *[C. Benavente]*
	Murcia, Hospital Universitario Virgen de la Arrixaca *[P. Iniesta]*
Sweden	Stockholm, Karolinska University hospital *[**E. Hellström-Lindberg**]*
	Göteborg, Sahlgrenska University Hospital *[H. Garelius]*
	Stockholm, Södersjukhuset *[M. Grövdal]*
	Umeå, Umeå regional hospital *[F. Lorenz]*

Country	**Participating centers** (City, Name organization, *[local investigator(s)]*)
	Halmstad, Teaching Hospital of Halmstad *[C. Karlsson]*<br>Linköping, University Hospital Linköping *[P. Antunovic / A. Jönsson]*<br>Luleå, Sunderby Hospital *[L. Brandefors]*<br>Örebro, Örebro University Hospital *[P.Kozlowski]*<br>Sundsvall, Sundsvalls sjukhus *[K. Larsson / J. Wallvik]*<br>Uppsala, Uppsala University *[E. Ejerblad]*<br>Eskilstuna, Mälarsjukhuset *[E. Hesse Sundin]*<br>Lund, Lund University Hospital *[L. Nilsson]*
United Kingdom	Leeds, Leeds Teaching Hospitals NHS Trust *[**<u>C. Cargo</u>** / M. Karakantza]*<br>Aberdeen, Aberdeen Royal Infirmary *[<u>D. Culligan</u>]*<br>Worcester, Worcestershire Acute Hospitals NHS Trust *[J. Mills]*<br>Blackpool, Blackpool Victoria Hospital *[S. Kolade / P. Cahalin]*<br>Steeton, Airedale NHS Trust *[E. Nga]*<br>Northampton, Northampton General Hospital *[J. Parker / A. Bowen]*<br>Harrogate, Harrogate District Hospital *[C. Hall]*<br>Bradford, Bradford Royal Infirmary *[S. Ackroyd]*<br>Bournemouth, Royal Bournemouth Hospital *[S. Killick]*<br>Wakefield, Mid Yorkshire Hospitals *[J. Ashcroft]*<br>Truro, Royal Cornwall Hospital *[D. Creagh]*<br>York, York Hospital *[L. Bond]*<br>Cambridge, Addenbrooke's Hospital *[C. Wong / A. Warren]*<br>Hull, Hull and East Yorkshire Hospitals NHS Trust *[S. Green / S. Ali]*<br>Oxford, John Radcliffe Hospitals NHS Trust *[P. Vyas]*<br>Newcastle upon Tyne, Royal Victoria Infirmary *[G. Jones]*<br>Nottingham, City Hospital *[R. Radia / E. Das-Gupta]*<br>Glasgow, Western Infirmary *[M. Drummond]*<br>Huddersfield, Huddersfield Royal Infirmary *[K. Rothwell]*<br>Birmingham, Queen Elizabeth Hospital *[C. Craddock]*<br>Manchester, Christie Hospital *[M. Dennis]*

National Principal Investigator are stated in **bold**; Steering Committee Member are <u>underlined</u>.

References

1. Garelius HKG, Johnston WT, Smith AG, Park S, de Swart L, Fenaux P, et al. Erythropoiesis-stimulating agents significantly delay the onset of a regular transfusion need in nontransfused patients with lower-risk myelodysplastic syndrome. J Intern Med. 2017 Mar;281(3):284–99.
2. Austin PC. The use of propensity score methods with survival or time-to-event outcomes: reporting measures of effect similar to those used in randomized experiments. Statistics in Medicine. 2013 Sep 30;33(7):1242.
3. Cole SR, Hernán MA. Adjusted survival curves with inverse probability weights. Comput Methods Programs Biomed. 2004 Jul;75(1):45–9.
4. Snapinn SM, Jiang Q, Iglewicz B. Illustrating the Impact of a Time-Varying Covariate with an Extended Kaplan-Meier Estimator. The American Statistician. 2005;59(4):301–7.
